# Supplementary material for: Transient and Partial Nuclear Lamina Disruption Promotes Chromosome Movement in Early Meiotic Prophase
Source: Dev Cell. 2018 Apr 23;45(2):212–225.e7. doi: 10.1016/j.devcel.2018.03.018 (PMC5920155; doi:10.1016/j.devcel.2018.03.018)
Supplement: Document S1. Figures S1–S6 and Tables S1–S3 [file mmc1.pdf]

Developmental Cell, Volume *45*

Supplemental Information

Transient and Partial Nuclear Lamina Disruption Promotes Chromosome Movement in Early Meiotic Prophase

Jana Link, Dimitra Paouneskou, Maria Velkova, Anahita Daryabeigi, Triin Laos, Sara Labella, Consuelo Barroso, Sarai Pacheco Piñol, Alex Montoya, Holger Kramer, Alexander Woglar, Antoine Baudrimont, Sebastian Mathias Markert, Christian Stigloher, Enrique Martinez-Perez, Alexander Dammermann, Manfred Alsheimer, Monique Zetka, and Verena Jantsch

Supplemental Information

Figure S1

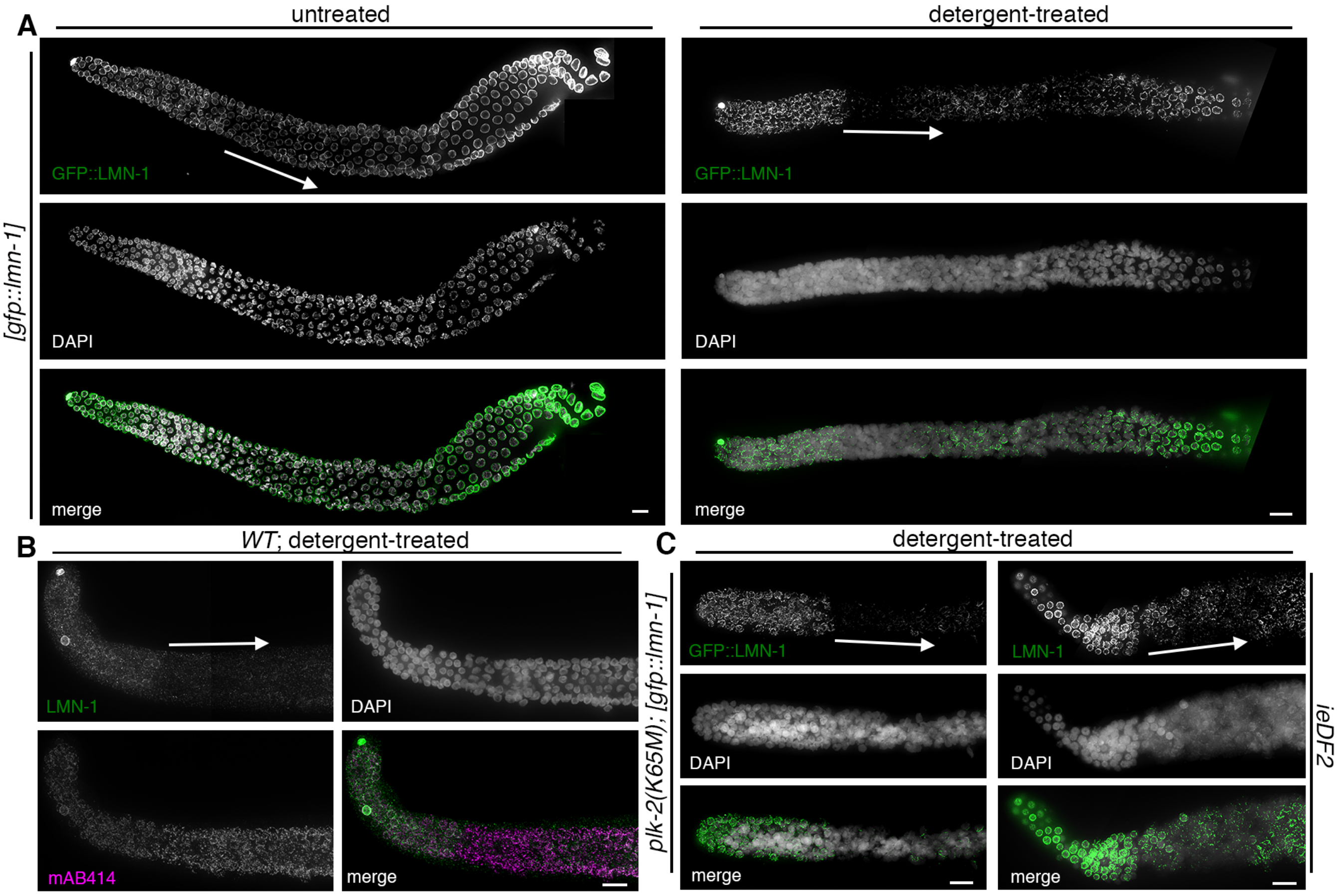

Figure S2

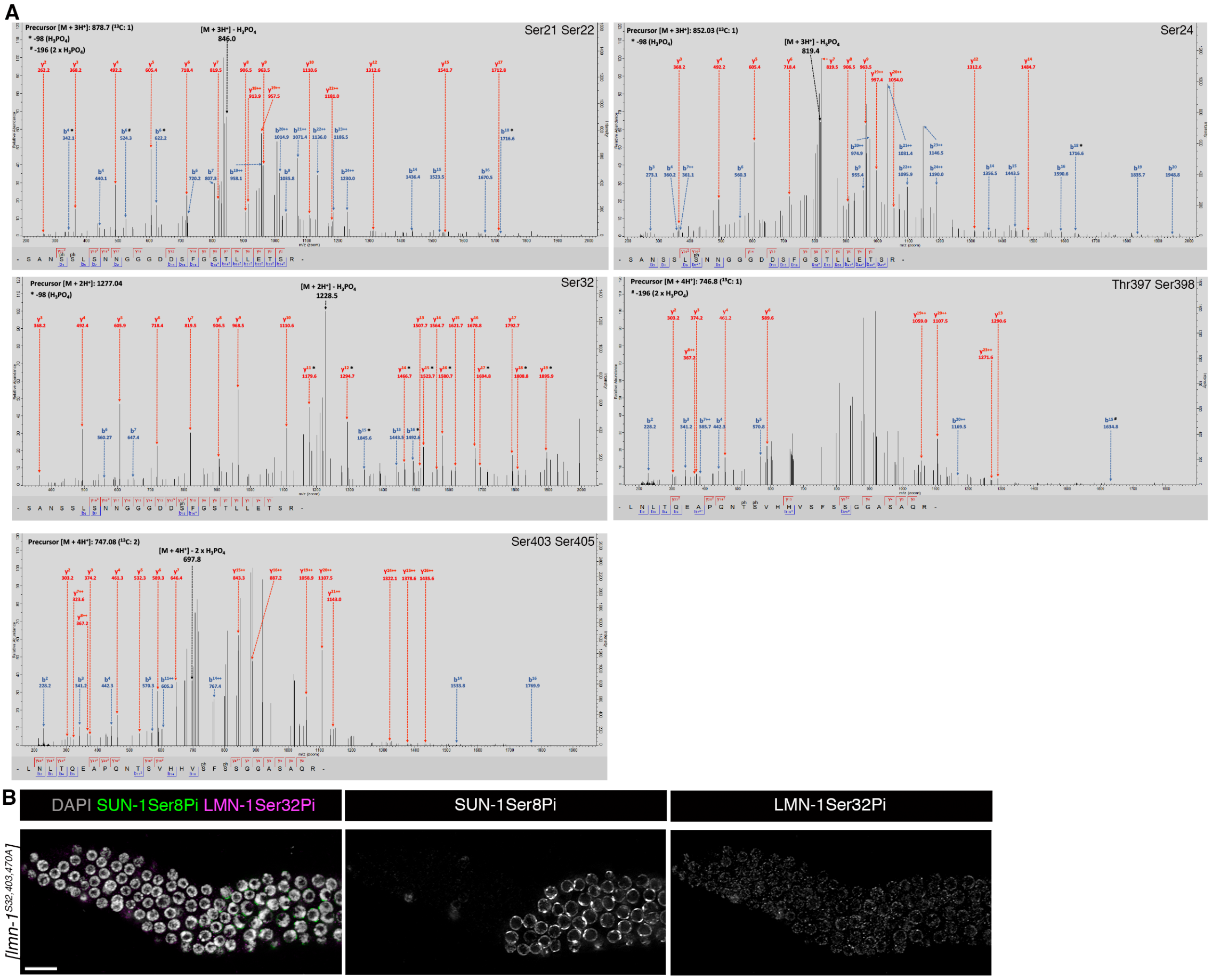
A
Ser21 Ser22
Ser24
Ser32
Thr397 Ser398
Ser403 Ser405
B
DAPI SUN-1Ser8Pi LMN-1Ser32Pi
SUN-1Ser8Pi
LMN-1Ser32Pi
[lmn-1S32,403,470A]

Figure S3

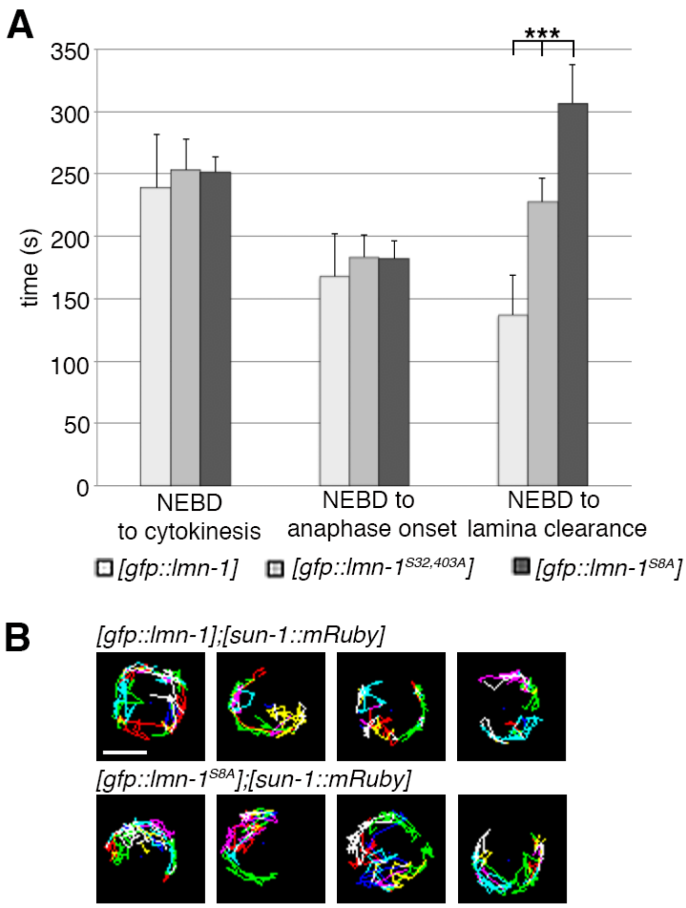
A
350
300
250
200
150
100
50
0
time (s)
***
NEBD
to cytokinesis
NEBD to
anaphase onset
NEBD to
lamina clearance
[gfp::lmn-1]
[gfp::lmn-1S32,403A]
[gfp::lmn-1S8A]
B
[gfp::lmn-1];[sun-1::mRuby]
[gfp::lmn-1S8A];[sun-1::mRuby]

Figure S4

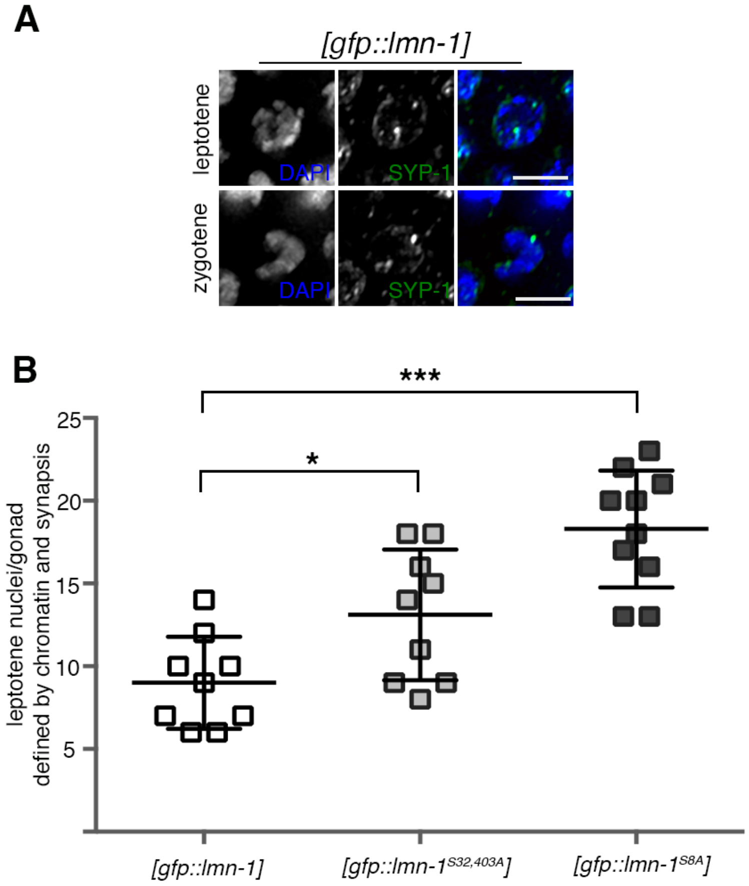
A
[gfp::lmn-1]
leptotene
zygotene
DAPI
SYP-1
B
***
*
leptotene nuclei/gonad defined by chromatin and synapsis
25
20
15
10
5
[gfp::lmn-1]
[gfp::lmn-1S32,403A]
[gfp::lmn-1S8A]

Figure S5

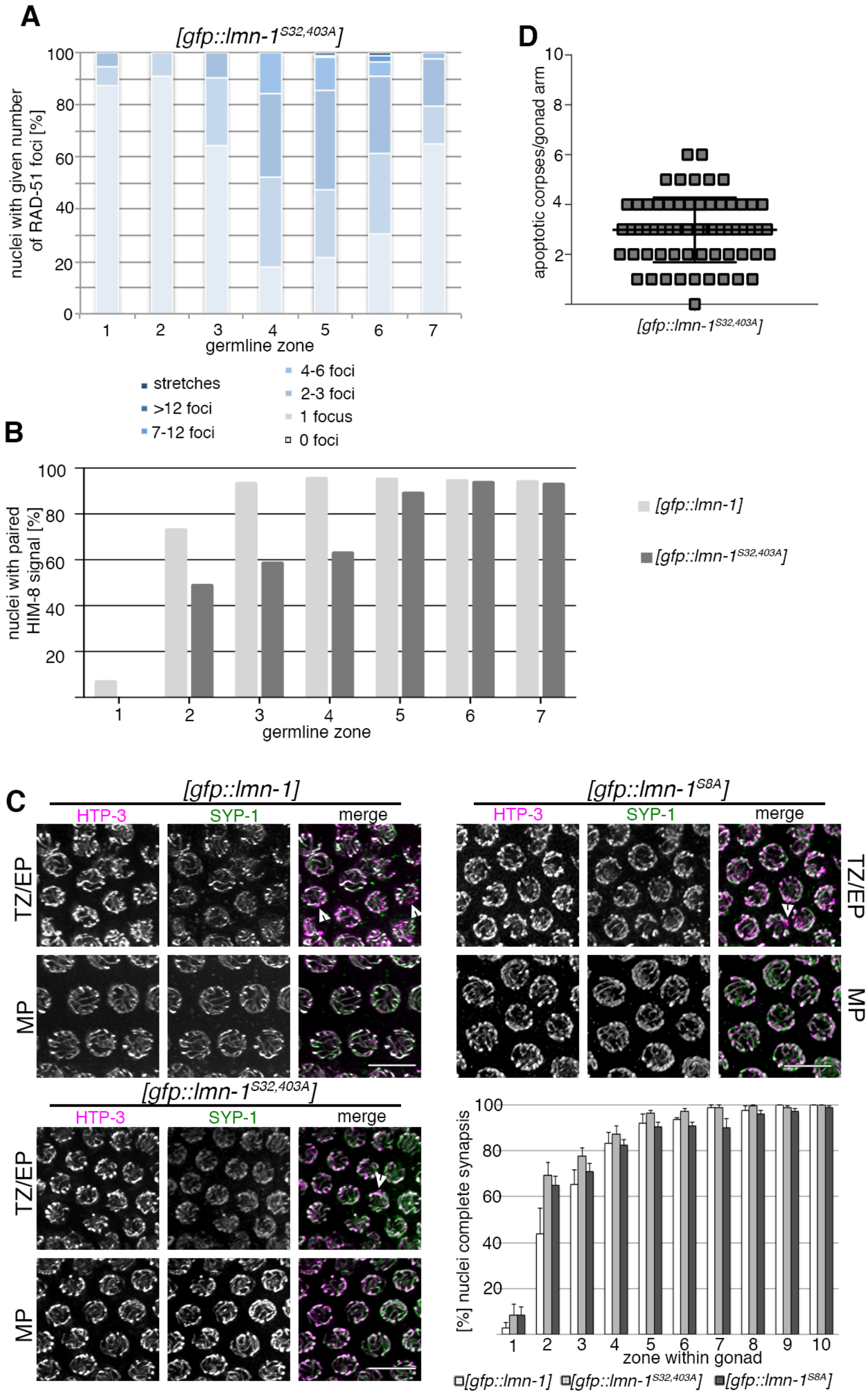
A
[gfp::lmn-1S32,403A]
nuclei with given number of RAD-51 foci [%]
0
20
40
60
80
100
1 2 3 4 5 6 7
germline zone
stretches
>12 foci
7-12 foci
4-6 foci
2-3 foci
1 focus
0 foci
B
nuclei with paired HIM-8 signal [%]
20
40
60
80
100
1 2 3 4 5 6 7
germline zone
[gfp::lmn-1]
[gfp::lmn-1S32,403A]
C
[gfp::lmn-1]
HTP-3
SYP-1
merge
TZ/EP
MP
[gfp::lmn-1S8A]
HTP-3
SYP-1
merge
TZ/EP
MP
[gfp::lmn-1S32,403A]
HTP-3
SYP-1
merge
TZ/EP
MP
[%] nuclei complete synapsis
20
40
60
80
100
1 2 3 4 5 6 7 8 9 10
zone within gonad
[gfp::lmn-1]
[gfp::lmn-1S32,403A]
[gfp::lmn-1S8A]
D
apoptotic corpses/gonad arm
2
4
6
8
10
[gfp::lmn-1S32,403A]

Figure S6

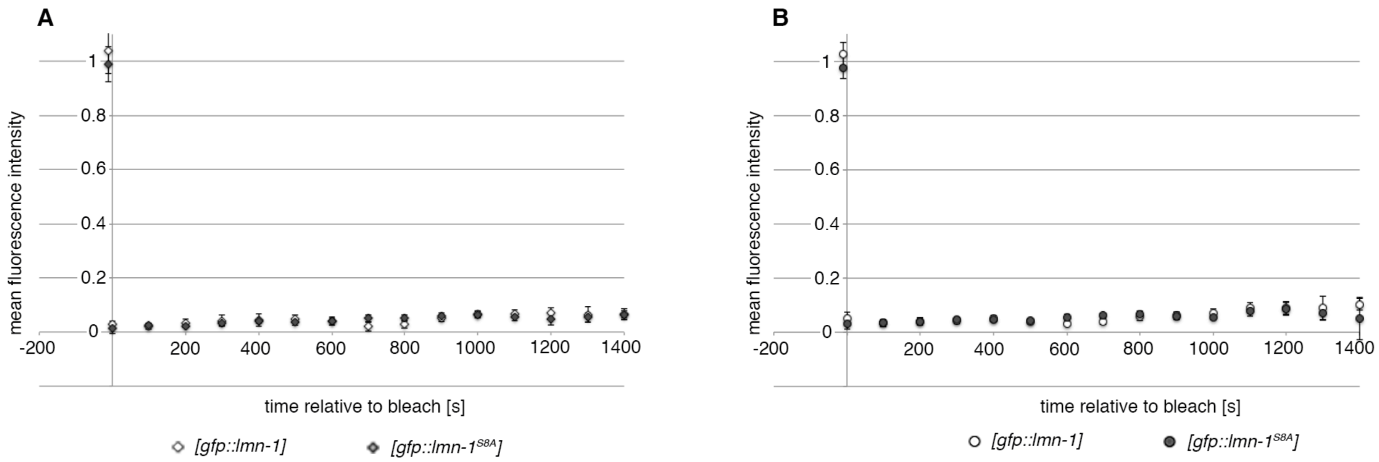
A
B
mean fluorescence intensity
1
0.8
0.6
0.4
0.2
0
-200
200
400
600
800
1000
1200
1400
time relative to bleach [s]
[gfp::lmn-1]
[gfp::lmn-1^S8A]

Figure S1

Decreased detergent resistance is specific to the lamina of early meiotic nuclei and is less prominent in *ieDF2* mutants. Related to Figure 1. (A) GFP::LMN-1 localization in untreated and detergent-treated wild-type gonads. The LMN-1 localization pattern in detergent-treated gonads is highly reproducible, reflecting changing detergent sensitivity as cells progress through the different prophase I stages. (B) Nuclear pore (mAB414) staining and LMN-1 localization pattern in detergent-treated wild-type gonads. (C) GFP::LMN-1 localization and LMN-1 immunofluorescence in detergent-treated *plk-2(K65M)* and *ieDF2* gonads. Arrows indicate the meiotic entry/transition zone; scale bars, 10 µm. Gonads shown in (A-C) consist of multiple maximum projection images stitched together to show larger sections of the gonads.

Figure S2

Identification of meiotic LMN-1 phosphorylation sites. Related to Figure 2. (A) Selected MS/MS fragmentation spectra showing phosphorylation sites on phosphopeptides corresponding to LMN-1 (Q21443) residues 18–42 (SANSSLSNNGGGDDSFGSTLLETSR) and 387–413 (LNLTQEAPQNTSVHHVSFSSGGASAQR). Annotated peaks correspond to ion species with a mass difference of −98 (*) or −196 (#), resulting from the loss of one or two phosphate moieties, respectively. Note that in the spectra showing Thr397 and Ser398 phosphorylation, the mass of ions Y19, Y20 and Y23 correspond to intact doubly phosphorylated fragments. (B) The LMN-1Ser32pi antibody specifically recognizes phospho-Ser32 LMN-1. LMN-1Ser32pi and SUN-1Ser8pi staining in transition zone of a mutant expressing Ser32A LMN-1. Scale bar, 10 µm. Gonads shown in (B) consist of multiple maximum projection images stitched together to show larger sections of the gonads.

Figure S3

Mutants expressing non-phosphorylatable LMN-1 have delayed lamina clearance after mitotic nuclear envelope breakdown (NEBD). Related to Figure 3. (A) Kinetics of the first mitotic embryonic division, showing the average periods between selected

hallmark mitotic events in the wild type and in two mutants expressing non-phosphorylatable LMN-1. *[gfp::lmn-1]*, *n* = 8 embryos; *[gfp::lmn-1^{S32,403A}]*, *n* = 6 embryos; *[gfp::lmn-1^{S8A}]*, *n* = 6 embryos. For timing from NEBD to lamina clearance: P = 9.04×10^{-5} (***) for *[gfp::lmn-1]* vs *[gfp::lmn-1^{S32,403A}]*, 8.05×10^{-7} (***) for *[gfp::lmn-1]* vs *[gfp::lmn-1^{S8A}]* and 6.77×10^{-4} (***) for *[gfp::lmn-1^{S32,403A}]* vs *[gfp::lmn-1^{S8A}]*. $P > 0.05$ (ns) not indicated. P values were calculated using the two-tailed Student's *t*-test. Error Bars represent SD. (B) Additional displacement tracks of wild type and non-phosphorylatable *lmn-1* mutants. Despite the significantly reduced velocity of SUN-1::mRuby aggregates, they show comparable exploration of the nuclear surface. Scale bar, 5 µm.

Figure S4

SYP-1 immunofluorescence (synapsis marker) shows that leptotene nuclei accumulate in the gonads of mutants expressing non-phosphorylatable LMN-1. Related to Figure 4. (A) Leptotene nuclei are characterized by spherical, non-polarized chromatin with aggregates or small stretches of SYP-1. Zygotene nuclei also show aggregates or stretches of SYP-1 but are distinguished by their non-spherical, polarized chromatin. Scale bar, 5 µm. (B) Quantification of leptotene nuclei in *[gfp::lmn-1]*, *[gfp::lmn-1^{S32,403A}]* and *[gfp::lmn-1^{S8A}]* gonads. *[gfp::lmn-1]*, *n* = 9 gonads; *[gfp::lmn-1^{S32,403A}]*, *n* = 9 gonads; *[gfp::lmn-1^{S8A}]*, *n* = 10 gonads. $P = 0.021$ (*), $P < 0.0001$ (***). P values were calculated using the two-tailed Student's *t*-test. Scatter plots indicate mean and SD.

Figure S5

Meiotic chromosome axis formation and synapsis are normal in all mutants expressing non-phosphorylatable LMN-1; for other meiotic markers, *[gfp::lmn-1^{S32,403A}]* has an intermediate phenotype between *[gfp::lmn-1]* and *[gfp::lmn-1^{S8A}]*. Related to Figure 5. (A) Kinetics of RAD-51 localization in the *[gfp::lmn-1^{S32,403A}]* germline (addition to Fig. 5B). *[gfp::lmn-1^{S32,403A}]*, *n* = 3 gonads. Wild-type values are identical to those in Fig. 5B. (B) Kinetics of X-chromosome pairing in the *[gfp::lmn-1^{S32,403A}]* germline (addition to Fig. 5C). *[gfp::lmn-1^{S32,403A}]*, *n* = 5 gonads. Wild-type

values are identical to those in Fig. 5C. *P* values were calculated using the Chi square test and equal to 0.0028 (**), <0.0001(****), <0.0001 (****), <0.0001 (****), 0.1564 (ns), 0.9116 (ns), 0.5281 (ns) for each germline zone, respectively. (C) Kinetics of meiotic synapsis formation. HTP-3 immunofluorescence marks both unsynapsed and synapsed chromosome axes; SYP-1 localizes only to synapsed chromosomes. Bottom right panel, proportion of nuclei in each zone showing complete SYP-1/HTP-3 colocalization (indicating completed synapsis). TZ/EP, transition zone/early pachytene cells; MP, mid-pachytene cells; arrowheads indicate regions with incomplete synapsis. *[gfp::lmn-1]*, *n* = 4 gonads; *[gfp::lmn-1^{S32,403A}]*, *n* = 7 gonads; *[gfp::lmn-1^{S8A}]*, *n* = 7 gonads. Error bars indicate SEM. Scale bars, 5μm. (D) Quantification of SYTO-12 positive corpses per gonad arm in the *[gfp::lmn-1^{S32,403A}]* mutant (addition to Fig. 5E). *[gfp::lmn-1^{S32,403A}]*, *n* = 67 gonads; *P* > 0.05 (ns) compared with *[gfp::lmn-1]* worms. *P* values were calculated using the Mann–Whitney *U*-test. Scatter plots indicate mean and SD.

Figure S6

FRAP analysis of LMN-1 in nuclei in the mitotic and the meiotic zones show no significant recovery. Related to Figure 3. (A) FRAP analysis of nuclei in the mitotic zone in *[gfp::lmn-1]* and *[gfp:lmn-1^{S8A}]* worms. The normalized mean intensity of GFP::LMN-1 is shown. Error bars indicate 90% confidence intervals. *[gfp:lmn-1], n* = 3 nuclei; *[gfp::lmn-1^{S8A}], n* = 5 nuclei. (B) FRAP analysis of meiotic nuclei in transition zone in *[gfp::lmn-1]* and *[gfp:lmn-1^{S8A}]* worms. The normalized mean intensity of GFP::LMN-1 is shown. Error bars indicate 90% confidence intervals. *[gfp:lmn-1], n* = 3 nuclei; *[gfp::lmn-1^{S8A}], n* = 3 nuclei.

Table S1: Viability and brood size of strains used in this study. Related to experimental models in STAR Methods.

Strain	% Viability +/- sd	Brood size +/- sd	n
lmn-1(tm1502);[gfp::lmn-1]	99.4% +/- 0.8	223.6 +/- 30.9	18
lmn-1(tm1502); [gfp::lmn-1^{S32,403A}]	99.2% +/- 0.7	230.1 +/- 39.5	18
lmn-1(tm1502); [gfp::lmn-1^{S8A}]	99.3% +/- 0.6	221.5 +/- 43.2	18
lmn-1(tm1502); [gfp::lmn-1]; ced-3(n717)	95.4% +/- 6.5	161.1 +/- 38.6	14
lmn-1(tm1502); [gfp::lmn-1^{S32,403A}]; ced-3(n717)	89.7% +/- 21.2	127.7 +/- 41.7	14
lmn-1(tm1502); [gfp::lmn-1^{S8A}]; ced-3(n717)	74.3% +/- 21.4	134.3 +/- 56.3	14
[sun-1^{S10A}::gfp]; sun-1(ok1282)[1]	55.29% +/- 13.3	135.15 +/- 29.6	20
lmn-1(tm1502);[gfp::lmn-1]; [sun-1::gfp]; sun-1(ok1282)	99.1% +/- 1.7	227.4 +/- 31.2	20
lmn-1(tm1502);[gfp::lmn-1]; [sun-1^{S10A}::gfp]; sun-1(ok1282)	57.6% +/- 18.4	158.2 +/- 45.8	20
lmn-1(tm1502); [gfp::lmn-1^{S8A}]; [sun-1^{S10A}::gfp]; sun-1(ok1282)	50.69% +/- 23.5	134.8 +/- 42.4	17
[sun-1^{S10A}::gfp] ced-3(n717); sun-1(ok1282)	46.9% +/- 13.8	128.2 +/- 47.2	13
lmn-1(tm1502);[gfp::lmn-1]; [sun-1::gfp] ced-3(n717); sun-1(ok1282)	95.5% +/- 0.9	156.0 +/- 48.5	7

[1] This is a single copy integration line on chromosome IV. We obtained the integration line with the same transgene on chromosome II with 88% viability. This difference is likely due to lower expression levels of the transgene when integrated on chromosome IV.

lmn-1(tm1502); [gfp::lmn-1^{S8A}]; [sun-1^{S10A}::gfp] ced-3(n717); sun-1(ok1282)	49.2 +/- 9.7	101.6 +/- 50.7	10
lmn-1(jf98[GFP::lmn-1])	68% +/- 15.97	182.5% +/- 63.68	10

Table S2: Parameters used for the EM analysis. Related to Figure 5.

cell stage	Nuclear diameter in EM [µm]	Cellular characteristics
mitotic	not analyzed	no polarised nucleolus
early prophase (transition zone)	2.5-3.5	polarised nucleolus<br>germline granules present
later prophase<br>(mid- late pachytene)	above 4.3	Frequent extended SCs<br>Ref. *

* Rog, O. et al; 2017 – mid/late prophase nuclei with intact, extended synapsis have an approx. diameter of 4.9 µm i.e. larger than what we analyzed

Table S3. Oligonucleotides used in this study. Related to STAR Methods.

REAGENT or RESOURCE	SOURCE	IDENTIFIER
Oligonucleotides		
Forward primer for cloning the lmn-1 5’ UTR into the P4-P1R entry vector<br>5’ GGGGACAACTTTGTATAGAAAAGTTGGGGAAAA ACTACCGCAAAAAACCA 3’	This paper	N/A
Forward primer for bridging the GFP with the 5’ UTR<br>5’ GCAGCGAGAAAAATGGTGAGCAAGGGCGAG 3’	This paper	N/A
Reverse primer for cloning the lmn-1 5’UTR merged with the GFP into the P4-P1R entry vector<br>5’ GGGGACTGCTTTTTTGTACAAACTTGGCTTGTACAGCTCGTCGTCCATG 3’	This paper	N/A
Reverse primer for cloning the lmn-1 5’ UTR into the P4-P1R entry vector<br>5’ GGGGACTGCTTTTTTGTACAAACTTGG TTTTCTCGCTGCTTTCTGAAGAG	This paper	N/A
Forward primer for cloning lmn-1 CDS into the pDONR 221 vector<br>5’ GGGGACAAGTTTGTACAAAAAAGCAGGCTCC ATGTCATCTCGTAAAGGTACTCGTA 3’	This paper	N/A
Reverse primer for cloning lmn-1 CDS into the pDONR 221 vector<br>5’ GGGGACCACTTTGTACAAGAAAGCTGG GTCTTACATGATGGAACAACGATCGG 3’	This paper	N/A
Forward primer for cloning the lmn-1 3’ UTR into the P2r-P3 entry vector<br>5’ GGGGACAGCTTTCTTGTACAAAGTGGGG TTGAGAGTCGATATTACATCAATCC 3’	This paper	N/A
Reverse primer for cloning the lmn-1 3’ UTR into the P2r-P3 entry vector<br>5’ GGGGACAACTTTGTATAATAAAGTTG CAACTTGATGTCCTGCCGAG 3’	This paper	N/A
Forward primer for introducing the S32A point mutation<br>5’ AGGAGGCGACGATGCTTTGT3’	This paper	N/A
Reverse primer for introducing the S32A point mutation<br>5’ ACAAAGCATCGTCGCCTCCT 3’	This paper	N/A
Forward primer for introducing the S403A point mutation<br>5’ TCATCACGTCGCTTTTTCATCC 3	This paper	N/A
Reverse primer for introducing the S403A point mutation<br>5’ GGATGAAAAAGCGACGTGATGA 3’	This paper	N/A
Forward primer for introducing the S470A point mutation<br>5’ AAGAAGAACAAGCTATCGGAGGAT 3’	This paper	N/A

Reverse primer for introducing the S470A point mutation<br>5’ ATCCTCCGATAGCTTGTTCTTCTT 3’	This paper	N/A
Forward primer for generating sgRNA1 for tagging lmn-1 with GFP<br>*5’* TCTTGGAGAGATGTAAGAGAAGAG *3’*	This paper	N/A
Reverse primer for generating sgRNA1 for tagging lmn-1 with GFP<br>5’ AAACCTCTTCTCTTACATCTCTCC 3’	This paper	N/A
Forward primer for generating sgRNA2 for tagging lmn-1 with GFP<br>5’ TCTTGTCTGAAGAGTTAATTATAT 3’	This paper	N/A
Reverse primer for generating sgRNA2 for tagging lmn-1 with GFP<br>5’ AAACATATAATTAACTCTTCAGAC 3’	This paper	N/A
Forward primer for generating sgRNA3 for tagging lmn-1 with GFP<br>5’ TCTTGGAAAAATGTCATCTCGTAA 3’	This paper	N/A
Reverse primer for generating sgRNA3 for tagging lmn-1 with GFP<br>5’ AAACTTACGAGATGACATTTTTCC 3’	This paper	N/A
Forward primer for generating the ds repair template for the GFP tagging of lmn-1<br>5’ CAAGAAATATCAAAATGTTGAGTCACGTTCGTGTGG<br>CGCCTATGACGTTTTCTCTCTTCTCTTACATCTCTCTT<br>TGTCTATTTCTGAAACTAAATTCAATATAATTAACTCTT<br>CAGAAAGCAGCGAGAAAAATGGTGAGCAAGGGCGAG 3’	This paper	N/A
Reverse primer for generating the ds repair template for the GFP tagging of lmn-1<br>5’ GCGACGAATTCGCTGAGCGCTCTAGCGTAACAATACGAGAACT<br>ACGAGTACCTTCACGAGGTGACATCTTGTACAGCTCGTCCATGC 3’	This paper	N/A
Forward primer for FISH probe amplification<br>5’ TACTTGGATCGGAGACGGCC 3’	(Silva et al., 2014)	N/A
Reverse primer for FISH probe amplification<br>5’ CTAACTGGACTCAACGTTGC 3’	(Silva et al., 2014)	N/A
