# Supplementary material for: Transient and Partial Nuclear Lamina Disruption Promotes Chromosome Movement in Early Meiotic Prophase
Source: Dev Cell. 2018 Apr 23;45(2):212–225.e7. doi: 10.1016/j.devcel.2018.03.018 (PMC5920155; doi:10.1016/j.devcel.2018.03.018)
Supplement: Document S2. Article plus Supplemental Information [file mmc2.pdf]

Developmental Cell

Article

Transient and Partial Nuclear Lamina Disruption Promotes Chromosome Movement in Early Meiotic Prophase

Graphical Abstract

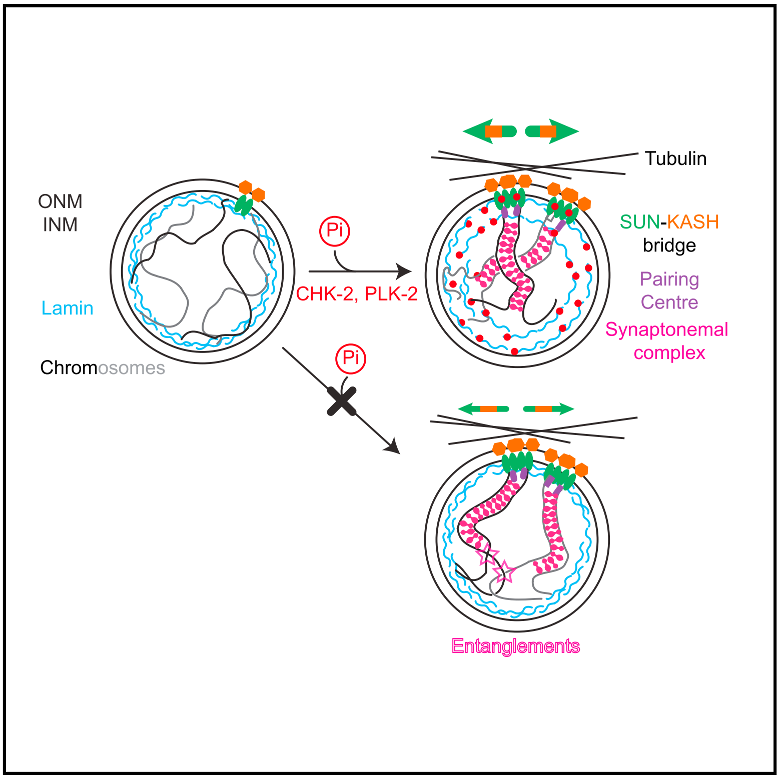

Highlights

- Upon meiotic entry, the protein network of the nuclear lamina is opened up/loosened
- Weakening of lamin crosslinks triggers dramatic chromatin reorganization
- Failure to remodel lamina crosslinking can lead to aberrant chromosome forms
- Meiotic phosphorylation of lamin is essential for maintaining chromosome integrity

Authors

Jana Link, Dimitra Paouneskou, Maria Velkova, ..., Manfred Alsheimer, Monique Zetka, Verena Jantsch

Correspondence

verena.jantsch@univie.ac.at

In Brief

The onset of chromosome movement within meiotic prophase nuclei is accompanied by lamina structural changes resembling those associated with mitotic nuclear envelope breakdown. Link, Paouneskou et al. show that meiotic lamina crosslink remodeling, through phosphorylation by kinases delivered via chromosome ends coupled to the nuclear envelope, ensures chromosome integrity.

Link et al., 2018, Developmental Cell 45, 212–225
April 23, 2018 © 2018 The Author(s). Published by Elsevier Inc.
https://doi.org/10.1016/j.devcel.2018.03.018

CellPress

Developmental Cell
Article

Transient and Partial Nuclear Lamina Disruption Promotes Chromosome Movement in Early Meiotic Prophase

Jana Link,[1,7] Dimitra Paouneskou,[1,7] Maria Velkova,[1] Anahita Daryabeigi,[1] Triin Laos,[2] Sara Labella,[3] Consuelo Barroso,[4] Sarai Pacheco Piñol,[4] Alex Montoya,[4] Holger Kramer,[4] Alexander Woglar,[1,8] Antoine Baudrimont,[1] Sebastian Mathias Markert,[6] Christian Stigloher,[6] Enrique Martinez-Perez,[4] Alexander Dammermann,[2] Manfred Alsheimer,[5] Monique Zetka,[3] and Verena Jantsch[1,9,*]

[1]Department of Chromosome Biology, Max F. Perutz Laboratories, University of Vienna, Vienna Biocenter, 1030 Vienna, Austria
[2]Department of Microbiology and Genetics, Max F. Perutz Laboratories, University of Vienna, Vienna Biocenter, 1030 Vienna, Austria
[3]Department of Biology, McGill University, 1205 Avenue Docteur Penfield, Montreal, QC H2A 1B1, Canada
[4]MRC Clinical Sciences Centre, Faculty of Medicine, Imperial College London, Du Cane Road, London W12 0NN, UK
[5]Department of Cell and Developmental Biology, Biocenter, University of Würzburg, Am Hubland, 97074 Würzburg, Germany
[6]Imaging Core Facility, Biocenter, University of Würzburg, Am Hubland, 97074 Würzburg, Germany
[7]These authors contributed equally
[8]Present address: Departments of Developmental Biology and Genetics, Stanford University School of Medicine, Stanford, CA 94305-5329, USA
[9]Lead Contact
*Correspondence: verena.jantsch@univie.ac.at
https://doi.org/10.1016/j.devcel.2018.03.018

SUMMARY

Meiotic chromosome movement is important for the pairwise alignment of homologous chromosomes, which is required for correct chromosome segregation. Movement is driven by cytoplasmic forces, transmitted to chromosome ends by nuclear membrane-spanning proteins. In animal cells, lamins form a prominent scaffold at the nuclear periphery, yet the role lamins play in meiotic chromosome movement is unclear. We show that chromosome movement correlates with reduced lamin association with the nuclear rim, which requires lamin phosphorylation at sites analogous to those that open lamina network crosslinks in mitosis. Failure to remodel the lamina results in delayed meiotic entry, altered chromatin organization, unpaired or interlocked chromosomes, and slowed chromosome movement. The remodeling kinases are delivered to lamins via chromosome ends coupled to the nuclear envelope, potentially enabling crosstalk between the lamina and chromosomal events. Thus, opening the lamina network plays a role in modulating contacts between chromosomes and the nuclear periphery during meiosis.

INTRODUCTION

To ensure faithful chromosome segregation at the first meiotic division, a physical tether needs to be established between parental chromosomes via crossover formation. This is achieved by repairing induced DNA double-strand breaks (DSBs) through homologous recombination, using a sister chromatid of the parental homolog as the repair template. This requires pairwise alignment of homologous chromosomes and chromosome pairing, stabilized by the synaptonemal complex (SC) (Zickler and Kleckner, 1998). These events take place during the extended prophase of meiosis I, which is characterized by rapid chromosome movements (Hiraoka and Dernburg, 2009; Koszul and Kleckner, 2009). Chromosome movement within the nucleus is driven by cytoskeletal forces generated in the cytoplasm and transmitted through the nuclear membranes via a conserved mechanism involving SUN (Sad1p, UNC-84) and KASH (Klarsicht, ANC-1, Syne Homology) domain proteins (*Caenorhabditis elegans* SUN-1 and ZYG-12 [Penkner et al., 2009; Sato et al., 2009]). These proteins are located in the inner and outer nuclear membranes, respectively, and physically interact in the lumen. Telomeres or subtelomeric repetitive regions (known as pairing centers [PCs] in *C. elegans*) initiate chromosome movement by coupling to the nuclear envelope (Woglar and Jantsch, 2014). These PCs are recruitment platforms for CHK-2 and polo kinases, which are needed to regulate chromosome movement, and are integral components of a surveillance mechanism that couples chromosome movement to synapsis and the establishment of crossovers through homologous recombination (Harper et al., 2011; Kim et al., 2015; Labella et al., 2011; Woglar et al., 2013). Vigorous back-and-forth chromosome movements aid the pairing and synapsis processes by eliminating topological entanglements and undesirable interchromosomal connections; this process is aberrant or delayed if movement is abrogated (Koszul and Kleckner, 2009). In *C. elegans*, chromosome movement prevents non-homologous synapsis and self-synapsis (Penkner et al., 2007; Rog et al., 2017). Thus, the lack of movement results in the formation of 12 unconnected univalents at diakinesis (instead of six connected bivalents) (Penkner et al., 2007). Chromosome movement coincides with drastic reorganization of the nuclear envelope. At this time, SUN-1 becomes hyperphosphorylated and concentrated at chromosome ends, which is

 © 2018 The Author(s). Published by Elsevier Inc.
This is an open access article under the CC BY license (http://creativecommons.org/licenses/by/4.0/).

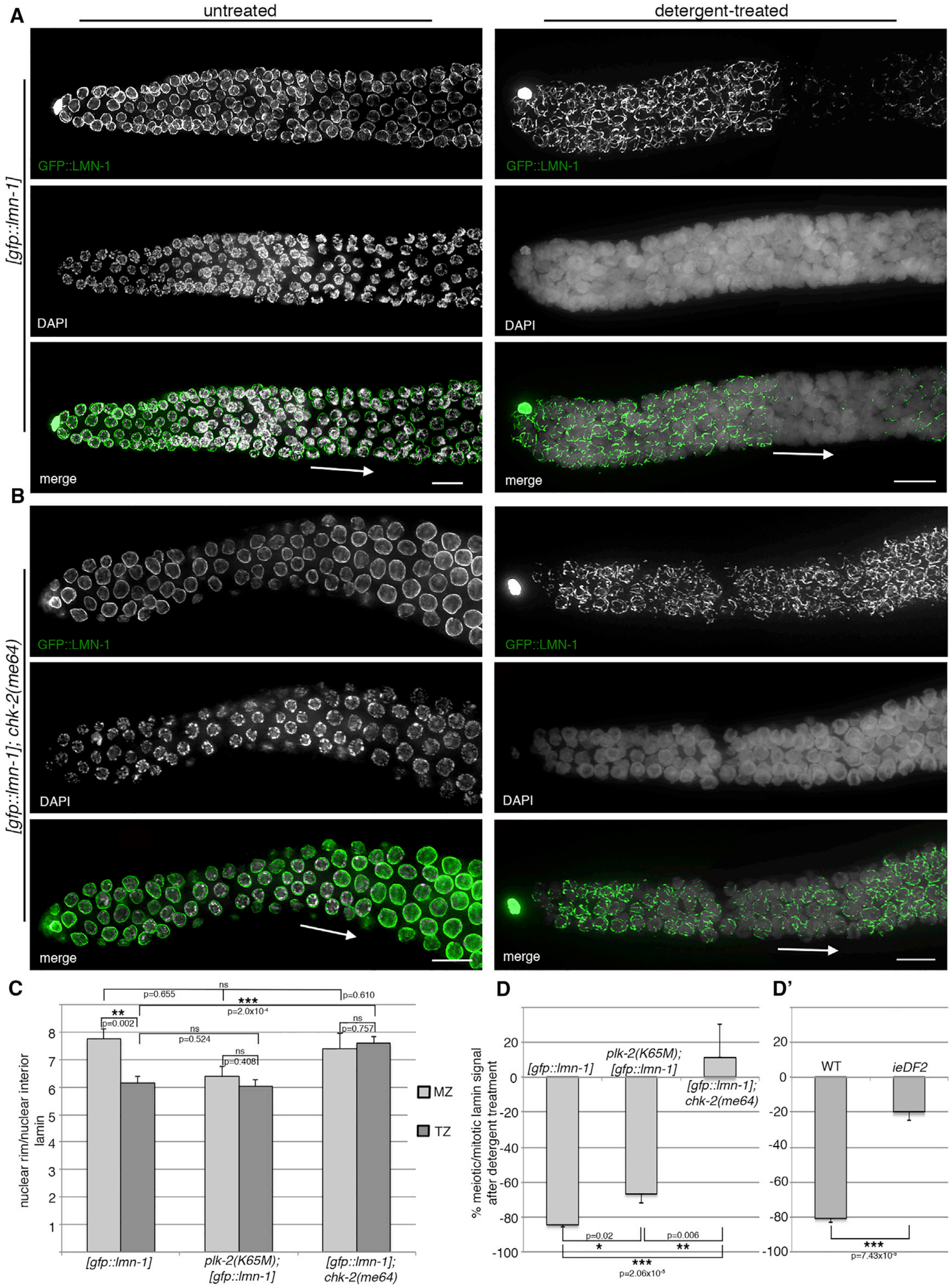

Figure 1. CHK-2-Dependent Changes in Nuclear Lamina Characteristics Occur at Meiotic Entry

(A and B) GFP::LMN-1 localization in the distal germline of untreated versus detergent-treated wild-type and *chk-2(me64)* gonads. Arrows highlight meiotic entry/transition zone. Scale bars, 10 μm. Gonads shown in (A and B) consist of multiple maximum projection images stitched together to show larger sections of the gonads.

(legend continued on next page)

mirrored by concentration of ZYG-12 and dynein. In response to aberrant chromosome transactions, SUN-1 hyperphosphorylation is part of a feedback mechanism that prolongs chromosome movement to resolve the problem (Woglar et al., 2013).

The nuclear lamina is a rigid protein network underlying the inner nuclear membrane and forms an integral part of the nuclear envelope. As well as providing nuclear stability, the lamina is also involved in DNA replication, transcription, and chromatin organization (Dechat et al., 2009). While vertebrates express different isoforms of A- and B-type lamins, the *C. elegans* genome encodes a single B-type isoform, *lmn-1* (Liu et al., 2000). All lamins are type V intermediate filaments composed of an N-terminal head, an α-helical coiled-coil rod domain, and a C-terminal tail domain (Stuurman et al., 1998). Lamins self-assemble to form higher-order structures (Heitlinger et al., 1991): *In vitro* assays revealed that lamins initially dimerize through their coiled-coil domains; dimers polymerize in a head-to-tail orientation; and antiparallel assembly of polymers form protofilaments, which combine to form 10-nm intermediate filaments (Foeger et al., 2006; Heitlinger et al., 1991; Karabinos et al., 2003). Lamin depolymerization is triggered by cyclin-dependant kinase-mediated phosphorylation of target sites adjacent to the coiled-coil domain (Gerace and Blobel, 1980; Heald and McKeon, 1990; Peter et al., 1990, 1991). This process is critical for nuclear envelope breakdown during mitosis, but has also been observed in interphase as a mechanism to "solubilize" the lamina (Kutay and Hetzer, 2008; Torvaldson et al., 2015). The nuclear lamina interacts with different nuclear components including chromatin and nuclear envelope proteins such as SUN-1, nuclear pores, and LEM (LAP2, emerin, Man1) domain proteins (Cohen-Fix and Askjaer, 2017), to form a three-dimensional, interconnected structure. This protein network could represent an obstacle to vigorous chromosomal movement and homologous pairing.

Very little is known about the lamin regulation during meiosis and how it contributes to chromosome organization, movement, pairing, and synapsis. Here, we used *C. elegans* as a model system to show that the nuclear lamina becomes "more soluble" upon meiotic entry and that this alteration is important for chromatin reorganization and the prevention of topologically unfavorable chromosome configurations.

RESULTS

Nuclear Lamina Characteristics Change after Meiotic Entry

To investigate whether characteristics of the meiotic lamina network change upon meiotic entry, we constructed *C. elegans* strains expressing functional GFP::LMN-1 from a MosSCI (*Mos1*-mediated single copy insertion) defined chromosomal insertion site in the *lmn-1* deletion mutant and tagged the endogenous locus by CRISPR. GFP::LMN-1 localization in both strains was identical to that of endogenous protein (see Table S1 for hatch rates and brood size). Extruded *C. elegans* gonads recapitulate the stages of meiotic prophase I in a temporal order (nuclei in the mitotic zone are followed by leptotene, zygotene, pachytene, diplotene, and diakinesis nuclei) (Hillers et al., 2017). After detergent extraction of dissected gonads, an abrupt loss of GFP::LMN-1 signal in nuclei at the start of the meiotic zone indicated a dramatic change in lamina organization (Figures 1A and S1A). In contrast, the lamina of nuclei residing in the mitotic region resisted detergent extraction. Detergent resistance was restored in nuclei at later stages of prophase I (mid-pachytene). We observed the same pattern by staining detergent-treated untagged LMN-1 in wild-type gonads with anti-LMN-1 antibody. Intriguingly, no such changes in detergent sensitivity were observed for nuclear pore complexes, suggesting that detergent treatment does not disrupt membrane integrity (Figure S1B).

A frequently cited model (Naetar et al., 2017) suggests the existence of a more soluble pool of nuclear lamin protein that is not associated with the filamentous network underlying the nuclear membrane. It is possible that lamins can exchange between the soluble nuclear pool and the population incorporated into the nuclear lamina. By measuring GFP::LMN-1 intensity at the nuclear rim and interior in single-plane confocal images of the germline, we found that the ratio of GFP::LMN-1 intensity at the nuclear rim to the nuclear interior was significantly different in nuclei in the mitotic zone from those in transition zone cells (corresponding to the leptotene/zygotene stage whereby chromatin adopts a half-moon shape; Figure 1A, arrow). The ratio was lower in nuclei in the early transition zone, indicating a marked decrease in the amount of lamin at the nuclear periphery (Figure 1C). This finding confirms that lamina remodeling occurs after meiotic entry.

Meiotic Lamina Remodeling Requires CHK-2 Kinase Delivery via Chromosome Ends Anchored to the Nuclear Periphery

Lamin has many post-translational modification sites (Simon and Wilson, 2013). We therefore wanted to examine whether LMN-1 modifications could be responsible for lamina remodeling in early prophase I. Thus, we performed the detergent extraction assay on *plk-2* and *chk-2* mutants expressing GFP::LMN-1 from the endogenous locus. In the *plk-2* mutant the catalytic domain is mutated (K65M), and both *plk-2* (data not shown) and *chk-2* mutants are defective in meiotic chromosome movement (Wynne et al., 2012). In *plk-2* mutant animals, most lamin could be extracted (Figures 1D and S1C). In contrast, in *chk-2* mutants the lamina resisted detergent extraction throughout meiotic prophase I (Figures 1B and 1D). Consistent with this

(C) GFP::LMN-1 intensity in mitotic zone (MZ) and transition zone (TZ) nuclei of wild-type and kinase mutants: ratio of signals at the nuclear periphery/nuclear interior. [*gfp*::*lmn-1*], n = 12 nuclei in each zone; *plk-2*(*K65M*);[*gfp*::*lmn-1*], n = 8 nuclei in each zone; *chk-2*(*me64*);[*gfp*::*lmn-1*], n = 8 nuclei in each zone, taken from 2–3 gonads. Error bars represent SEM.

(D) GFP::LMN-1 intensity at the nuclear periphery in detergent-treated gonads of the wild-type and kinase mutants: meiotic nuclear signal as percentage of the nuclear signal in the mitotic zone. [*gfp*::*lmn-1*], n = 5 gonads (at least 20 mitotic zone and 8 meiotic nuclei analyzed in each); *plk-2*(*K65M*);[*gfp*::*lmn-1*], n = 5 gonads (at least 20 mitotic zone and 10 meiotic nuclei analyzed in each); *chk-2*(*me64*);[*gfp*::*lmn-1*], n = 3 gonads (at least 15 mitotic zone and 15 meiotic nuclei analyzed in each). Error bars represent SEM.

(D′) LMN-1 immunofluorescence intensity in wild-type and *ieDF2* (bearing a deletion in the operon encoding pairing center proteins). Wild-type, n = 4 gonads; *ieDF2*, n = 4 gonads; at least 15 nuclei from the mitotic zone and 15 meiotic nuclei were analyzed in each gonad. Error bars represent SEM.

p values where calculated using the two-tailed Student's t test. *p < 0.05, **p < 0.01, ***p < 0.001. See Figure S1 for additional data.

finding, the ratio of GFP::LMN-1 intensity at the nuclear rim to nuclear interior was similar in mitotic and meiotic nuclei in the *chk-2* mutant (Figure 1C). The difference in values for this ratio between mitotic and meiotic nuclei was also much less pronounced in *plk-2* mutants. In contrast to the wild-type, in absence of PLK-2 or CHK-2, LMN-1 was not significantly reduced at the nuclear lamina at meiotic onset. Thus, in the absence of these meiotic master regulatory kinases, lamin accumulates at the nuclear rim upon meiotic entry, despite normal meiotic entry.

In *C. elegans*, one end of each chromosome contains a subtelomeric repeat, the site of PC protein recruitment. These proteins in turn serve as recruitment platforms for CHK-2 and polo kinases and are essential for coupling chromosomes to the movement apparatus (Harper et al., 2011; Labella et al., 2011). At PCs, these kinases mediate the feedback control mechanism activated by events on individual chromosomes (such as synapsis) to reinforce chromosome end-led movement driven by SUN/KASH bridging (Woglar et al., 2013). In the *ieDF2* mutant, deletion of the operon encoding PC proteins abrogates PLK-2 and CHK-2 recruitment, and subsequently PC function. Intriguingly, LMN-1 is similarly resistant to detergent extraction as in the *chk-2* mutant (Figures 1D′ and S1C).

These findings reveal that CHK-2 and, to a lesser extent, PLK-2 are required for the remodeling of the nuclear lamina at meiotic onset, and that these kinase activities are mainly delivered or regulated by PC chromosome regions.

Phospho-LMN-1 Is Upregulated upon Meiotic Entry

We hypothesized that LMN-1 might be a direct target of meiotic kinase activity. Independent phospho-enrichment experiments revealed eight relevant LMN-1 phospho-modified sites within two clusters located directly before and directly after the central coiled-coil domain: Ser21, Ser22, Ser24, and Ser32; and Thr397, Ser398, Ser403, and Ser405 (Figures 2A and S2A). None of these sites are contained within a CHK-2 kinase motif; however, Ser21, Ser22, and Ser32 are predicted as polo kinase target sites *in silico*. Ser22 and Ser405 are found in a polo box binding domain. As these regions are involved in higher-order lamina polymerization (Güttinger et al., 2009), modifications at these sites might alter lamina network characteristics.

To further investigate the temporal and spatial pattern of site-specific LMN-1 phosphorylation, we raised a phospho-specific antibody against LMN-1Ser32 (Figures 2B and S2B). Strikingly, cytological analyses revealed that LMN-1Ser32 phosphorylation is specifically upregulated at the onset of meiosis (Figure 2B), indicated by overlapping positive SUN-1Ser8pi staining of nuclei. SUN-1Ser8pi labeling of the nuclear envelope in transition zone nuclei correlates with the period of meiotic entry and chromosome movement (Penkner et al., 2009). Interestingly, LMN-1 phosphorylation (at least in a fraction of LMN-1 molecules) occurs in the same germline region in which the lamina is more vulnerable to detergent extraction.

To determine which kinases are responsible for LMN-1 phosphorylation, we stained *plk-2* and *chk-2* mutants with the LMN-1Ser32pi antibody. The signal is absent in *chk-2* meiotic nuclei, but a residual, albeit much weaker, signal is present in *plk-2* transition zone nuclei (Figure 2B). This finding indicates that PLK-2 contributes to, but CHK-2 is essential for, meiosis-specific LMN-1Ser32 phosphorylation. Owing to kinase redundancy in the germline (Harper et al., 2011; Labella et al., 2011), PLK-1 might be responsible for the residual signal in *plk-2* mutants. LMN-1Ser32pi staining of *spo-11* and *ieDF2* mutants showed that meiotic LMN-1 phosphorylation is independent of DNA DSB induction but dependent on the presence of PCs (Figure 2B). This result, together with altered resistance to detergent extraction in the *ieDF2* mutant, suggests that PCs are essential for delivering active CHK-2/PLK-2 kinases to the nuclear envelope to mediate meiosis-specific lamin phosphorylation.

Multiple LMN-1 Phosphorylations Are Required for Meiotic Lamina Remodeling

Of the multiple LMN-1 phosphorylation sites we identified, we wanted to know whether phosphorylation of all or only a subpopulation is necessary for meiotic lamina remodeling. We therefore used the MosSCI system to construct GFP::LMN-1-expressing strains with single or multiple target serine or threonine sites substituted for non-phosphorylatable alanine residues, and a corresponding control strain expressing wild-type GFP::LMN-1 from the same chromosomal insertion locus (Frøkjaer-Jensen et al., 2008) (Figure 2A and Table S1). All constructs were expressed via the endogenous lamin promoter and introduced into the *lmn-1*(*tm1502*) mutant background. The (*tm1502*) allele bears a large, lethal deletion on the *lmn-1* locus (Haithcock et al., 2005; Mattout et al., 2011). In the [*gfp*::*lmn-1*^{S32,S403A}] mutant, one residue before and one residue after the coiled-coil domain are substituted for non-phosphorylatable alanine residues; in the [*gfp*::*lmn-1*^{S8A}] mutant, all eight phosphorylated residues are changed to alanine. Both *lmn-1* alleles fully complemented the *lmn-1*(*tm1502*) deletion (Table S1). Monitoring the first embryonic division in these strains (Figures 3A and S3A) revealed wild-type division characteristics regarding timing and chromosome behavior. Nuclear envelope permeabilization, assessed by monitoring equilibration of free nuclear histone: mCherry with the cytoplasm, also occurred in a timely manner. However, nuclear lamina disassembly following nuclear envelope breakdown was delayed, indicating altered lamina organization (with [*gfp*::*lmn-1*^{S32,403A}] and [*gfp*::*lmn-1*^{S8A}] behaving as an allelic series; Figure S3A).

We next investigated the meiotic lamina properties of the two non-phosphorylatable *lmn-1* mutants using the detergent extraction assay and measured GFP::LMN-1 intensity in the nuclear rim versus the nuclear interior (Figures 3B–3D). Ser32 and Ser403 substitution alone did not significantly affect the solubilization pattern of the meiotic nuclear lamina. However, substitution of all eight phosphorylation sites rendered the lamina resistant to detergent; this effect was comparable with that in the *chk-2* and *ieDF2* mutants (Figures 3B and 3C). Opposite to wild-type, the ratios of GFP::LMN-1 intensity at the nuclear rim versus the nuclear interior were similar for nuclei in the mitotic and meiotic zones in both non-phosphorylatable *lmn-1* mutants. In this assay, substitution of only Ser32 and Ser403 already resulted in decreased amounts of LMN-1 relocating from the rim to the nuclear interior (Figure 3D), suggesting that the assay of lamin quantification at the rim is more sensitive to the phospho status. This result supports the notion that phosphorylation sites flanking the coiled-coil domain are required for lamina reorganization in meiosis, resulting in a reduction in the amount of lamin associated with the nuclear envelope.

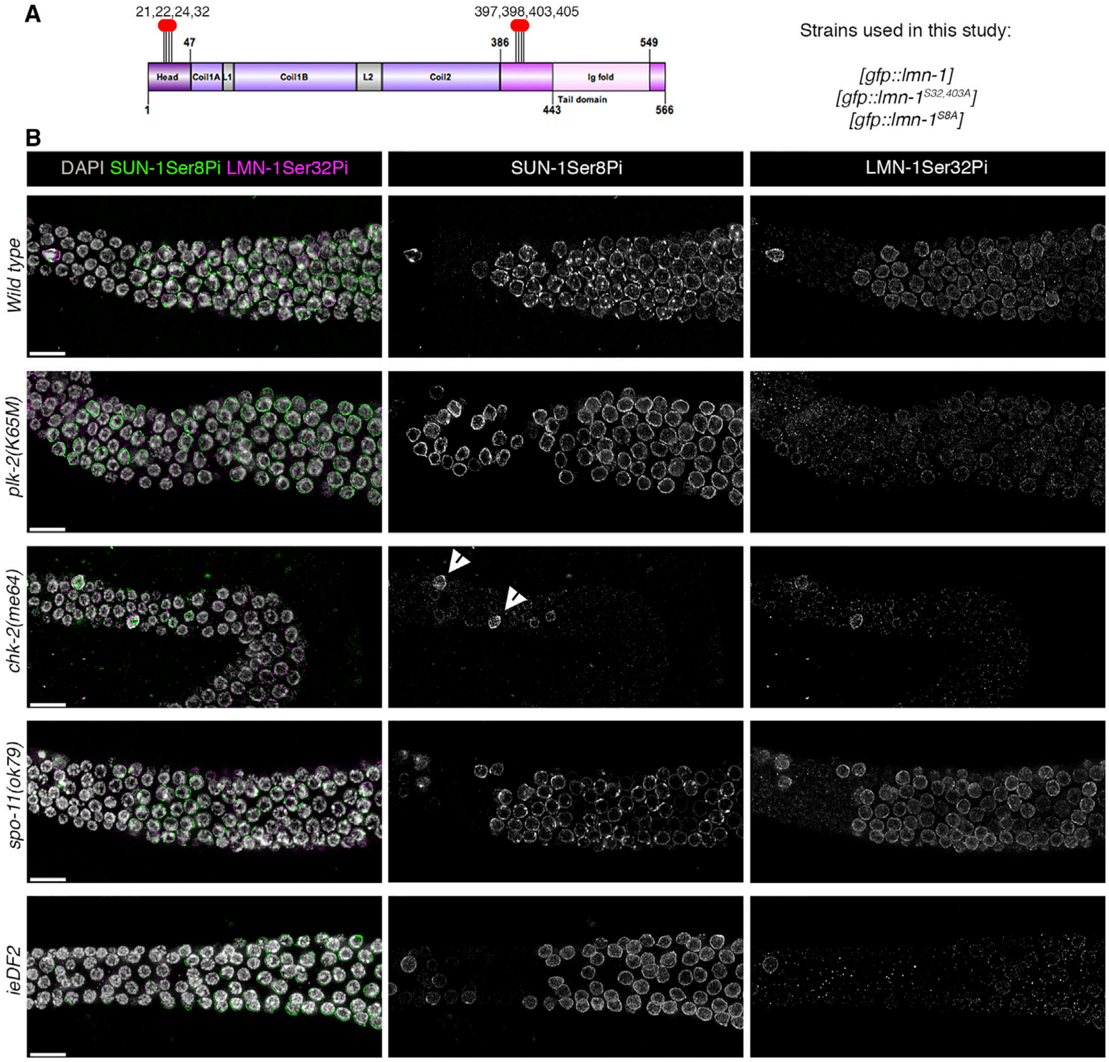

Figure 2. LMN-1 Ser32 Phosphorylation Specifically Occurs at Meiotic Onset, and Is PLK-2- and CHK-2-Dependent and Independent of DNA DSBs

(A) Schematic representation of LMN-1, indicating phosphorylation sites (red circles) relevant to this study. Numbers indicate amino acid positions. Strains generated for this study express wild-type GFP::LMN-1 or GFP::LMN-1 containing point mutations altering serine/threonine residues to non-phosphorylatable alanine. In one mutant strain, serines 32 and 403 were changed to alanine; in the S8A mutant, Ser21, Ser22, Ser24, Ser32, Thr397, Ser398, Ser403, and Ser405 were changed to alanine residues (see Table S1 for viability counts). The schematic representation of the protein was designed using the software GPS-SUMO sp 2.0.

(B) Localization of LMN-1Ser32pi and SUN-1Ser8pi signals in the transition zone. The SUN-1Ser8pi signal specifically marks meiotic entry. Representative images are shown for the wild-type and *plk-2*, *chk-2*, *spo-11*, and *ieDF2* mutants. Note the absence of meiotic phosphorylation at both SUN-1 Ser8 and LMN-1 Ser32 in the *chk-2* mutant. Arrowheads indicate phospho-modified mitotic nuclei. Scale bars, 10 μm. Gonads shown in (B) consist of multiple maximum projection images stitched together to show larger sections of the gonads.

See Figure S2 for additional data.

Interfering with Lamina Reorganization Affects Meiotic Chromosome Movement

Nuclei undergoing lamina phospho-modifications and remodeling in prophase I are located in the early transition zone; in the same region, vigorous microtubule-directed chromosome end movement mediated through SUN/KASH bridges occurs (Penkner et al., 2009; Sato et al., 2009). Therefore, we asked whether nuclear lamina remodeling is required for chromosome shuffling. Hence, we measured the speed of SUN-1::mRuby aggregate (marking chromosome ends) motion in the wild-type and [*gfp*::*lmn-1*^{S8A}] (Figure 3E). Interestingly, SUN-1 aggregates moved significantly slower in [*gfp*::*lmn-1*^{S8A}] than in control nuclei. Nevertheless, the SUN-1 aggregates still extensively explored the nuclear surface, and displayed splitting and fusion events (probably reflecting chromosome encounters and separations; Figure S3B). The reduced chromosome movement was sufficient for chromosome pairing and synapsis, suggesting that strong cytoskeletal forces can overcome the resistance due to the unmodified nuclear lamina, possibly caused by its increased rigidity. In contrast, movement-defective mutants display embryonic lethality and univalents at diakinesis (Penkner et al., 2007). To confirm that an unmodified lamina opposes

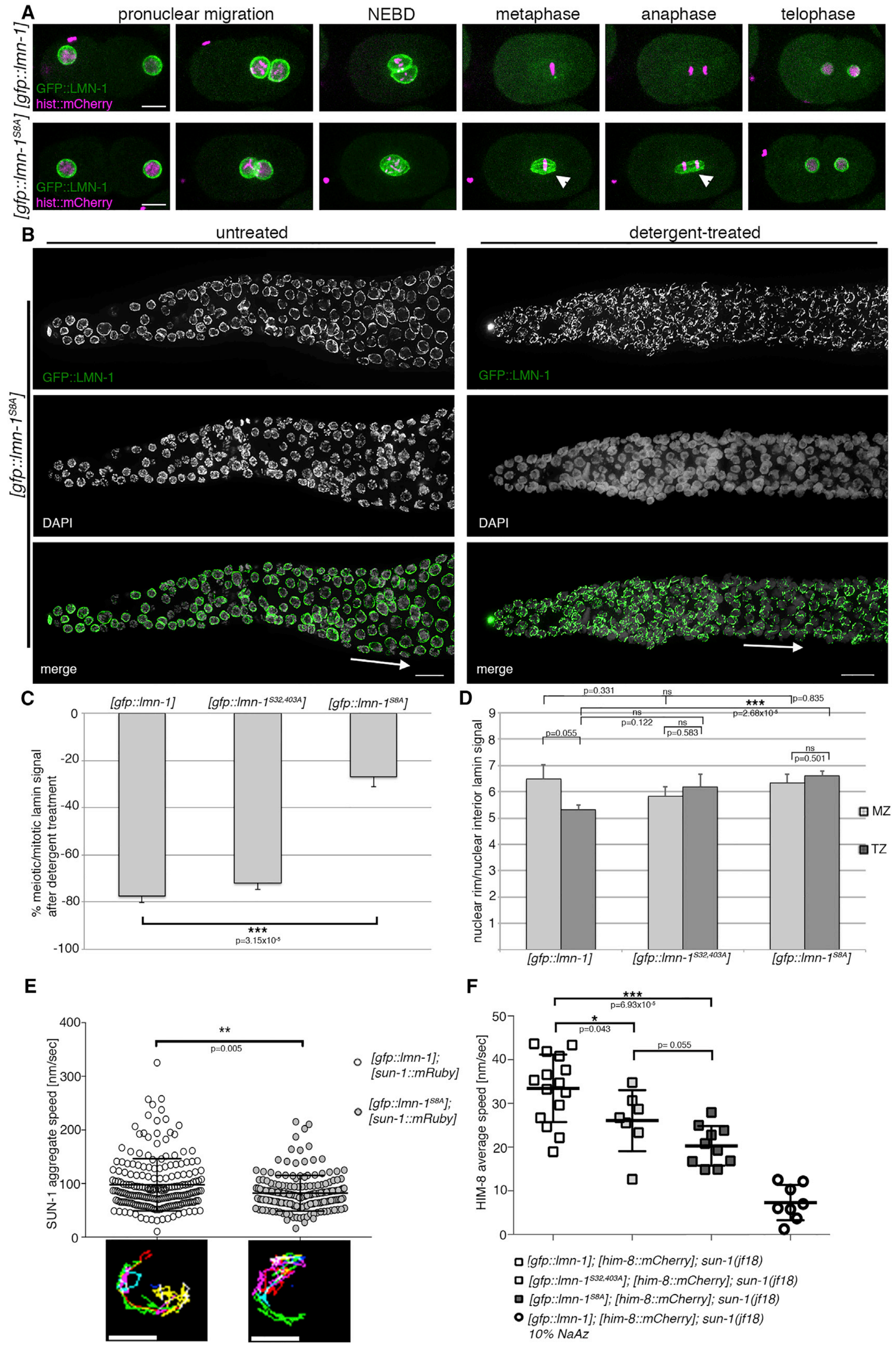

(legend on next page)

chromosome mobility, we tracked movement of the X chromosome end using HIM-8::mCherry in the *sun-1*(*jf18*) mutant. Transmission of cytoskeletal forces is disrupted in this mutant, although chromosome ends remain attached to the nuclear envelope (Penkner et al., 2009). This analysis supported our hypothesis that chromosome ends in the non-phosphorylatable *lmn-1* mutants have restricted mobility (Figure 3F). Furthermore, it confirmed that substituting only Ser32 and Ser403 confers a partial phenotype, and that [*gfp*::*lmn-1*^{S8A}] is a stronger allele.

Overall, these data provide evidence that the physicochemical properties of the nuclear lamina alter meiotic chromosome movement.

Animals Expressing Non-phosphorylatable LMN-1 Have Delayed Meiotic Entry/Leptotene Progression and Altered Heterochromatin Organization

Due to the observed decreased speed of SUN-1 aggregate movement, we aimed to investigate effects of non-phosphorylated LMN-1 on the progression through the earliest meiotic stages in more detail. Zygotene nuclei with half-moon-shaped chromatin (Hillers et al., 2017) are preceded by nuclei with a spherical chromatin distribution during meiotic entry, characterized by SC markers present in polycomplexes (Jantsch et al., 2007), and leptotene nuclei, positive for phospho-SUN-1 and unpolymerized SC components (Mlynarczyk-Evans and Villeneuve, 2017; Penkner et al., 2009). We hypothesize that chromatin has to undergo massive reorganization at meiotic entry/leptotene to achieve the crescent-shaped configuration and allow homologous chromosomes to align; in particular, chromatin needs to be removed from the nuclear periphery. Using SUN-1 phosphorylation and SC components as markers, we quantified nuclei undergoing chromatin reorganization and found a significant accumulation of nuclei with characteristics of delayed chromatin reorganization in [*gfp*::*lmn-1*^{S8A}] germlines (Figures 4A, 4B, and S4). These nuclei were positive for phospho-SUN-1 and had unpolymerized SC components but lacked the characteristic clustered chromatin shape.

To gain more detailed insight into chromatin reorganization, we additionally analyzed early prophase nuclei by electron microscopy. Comparison of both *lmn-1* mutants with the wild-type revealed a difference in heterochromatin distribution, as inferred from electron-dense clouds in areas of chromatin (Figures 4C and 4D; Table S2). Whereas wild-type nuclei had many small electron-dense heterochromatin patches, both *lmn-1* alleles displayed a significantly smaller number of large patches. The overall amount of heterochromatin in the nuclear sections was identical between strains. Heterochromatin localization within the nucleus also differed drastically between strains. Moreover, in both *lmn-1* mutants, most heterochromatic areas were in direct contact with the nuclear periphery. In contrast, in the wild-type many electron-dense areas were present in the nuclear interior, detached from the periphery.

Altogether, accumulation of meiotic entry/leptotene nuclei (a stage where we infer that chromatin removal from the periphery occurs), aberrant heterochromatin distribution in early prophase nuclei and reduced speed of SUN-1 aggregate movement suggest that the altered lamina properties in the mutant impair meiotic chromatin reorganization.

Expression of Non-phosphorylatable LMN-1 Causes Aberrant Recombination Intermediates, Occasional Unpaired Chromosomes, and Apoptotic Culling of Defective Oocytes

To investigate the consequences of altered chromosome movement and chromatin reorganization in mutants expressing non-phosphorylatable LMN-1, we analyzed meiotic progression in more detail (Figures 5A–5D and S5). DNA DSB induction and repair can be monitored by quantification of RAD-51 kinetics (Hillers et al., 2017). In addition, the proportion of SUN-1Ser8pi-positive germline is a readout for ongoing meiotic recombination and SC formation (Woglar et al., 2013), while

Figure 3. Phosphorylation of Multiple LMN-1 Residues Is Required for Lamina Remodeling and Efficient Chromosome End Movement in Early Meiosis but Dispensable for Mitosis

(A) Representative still images of the first mitotic division in early embryos: histone:mCherry marks mitotic chromosomes; GFP::LMN-1 indicates LMN-1. Note the lack of complete lamina disassembly during late metaphase and anaphase, with LMN-1 localizing to the remnants of the nuclear envelope separating maternal and paternal chromosome masses (arrowheads). Nevertheless, embryos are fully viable (see Table S1). For quantifications, see Figure S3A. Scale bars, 10 μm.

(B) Localization of GFP::LMN-1 in the distal germline of untreated and detergent-treated [*gfp*::*lmn-1*^{S8A}] gonads. In this mutant, all phosphorylation sites flanking the coiled-coil domain have been changed to non-phosphorylatable residues. Arrows indicate the onset of meiosis. Scale bars, 10 μm. Gonads shown in (B) consist of multiple maximum projection images stitched together to show larger sections of the gonads.

(C) GFP::LMN-1 intensity in detergent-treated gonads of the wild-type, [*gfp*::*lmn-1*^{S32,403A}], and [*gfp*::*lmn-1*^{S8A}] mutants: the meiotic nuclear signal is presented as a percentage of the nuclear signal in the mitotic zone. [*gfp*::*lmn-1*], n = 5 gonads (at least 20 mitotic zone and 8 meiotic nuclei analyzed in each gonad); [*gfp*::*lmn-1*^{S32,403A}], n = 5 gonads (at least 22 mitotic zone and 9 meiotic nuclei analyzed in each gonad); [*gfp*::*lmn-1*^{S8A}], n = 5 gonads (at least 15 mitotic zone and 10 meiotic nuclei analyzed in each gonad). Non-significant differences ($p > 0.05$) are not indicated. Error bars represent SEM.

(D) GFP::LMN-1 intensity in mitotic zone (MZ) and transition zone (TZ) nuclei of the wild-type and the two non-phosphorylatable *lmn-1* mutants: ratio of signals at the nuclear periphery/nuclear interior. [*gfp*::*lmn-1*], n = 16 nuclei in each zone; [*gfp*::*lmn-1*^{S32,403A}], n = 16 nuclei in each zone; [*gfp*::*lmn-1*^{S8A}], n = 16 nuclei in each zone. For all genotypes analyzed nuclei were taken from four different gonads. p values were calculated using the two-tailed Student's t test. Error bars represent SEM.

(E) Tracking of individual SUN-1::mRuby aggregates in transition zone nuclei of worms expressing wild-type or non-phosphorylatable LMN-1. Upper panel: the speed of individual SUN-1 aggregates from 14 nuclei/genotype is shown (wild-type, n = 207 aggregates; [*gfp*::*lmn-1*^{S8A}], n = 178 aggregates). Lower panel: projected displacement tracks for SUN-1 aggregates in representative nuclei of both genotypes. p value was calculated using the Mann-Whitney U test. Scatter plots indicate the mean and SD. For additional examples, see Figure S3B. Scale bars, 5 μm.

(F) Tracking of individual HIM-8::mCherry aggregates in early transition zone nuclei of worms expressing wild-type (n = 15 nuclei) and non-phosphorylatable LMN-1 in the *sun-1*(*jf18*) mutant ([*gfp*::*lmn-1*^{S32,403A}], n = 7 nuclei; [*gfp*::*lmn-1*^{S8A}], n = 10 nuclei). In this mutant, the cytoskeletal forces responsible for directed chromosome movement are eliminated. Background Brownian motion was assessed in worms mounted in 10% Na azide (n = 8 nuclei). p values were calculated using the two-tailed Student's t test. Scatter plots indicate the mean and SD.

*$p < 0.05$, **$p < 0.01$, ***$p < 0.001$.

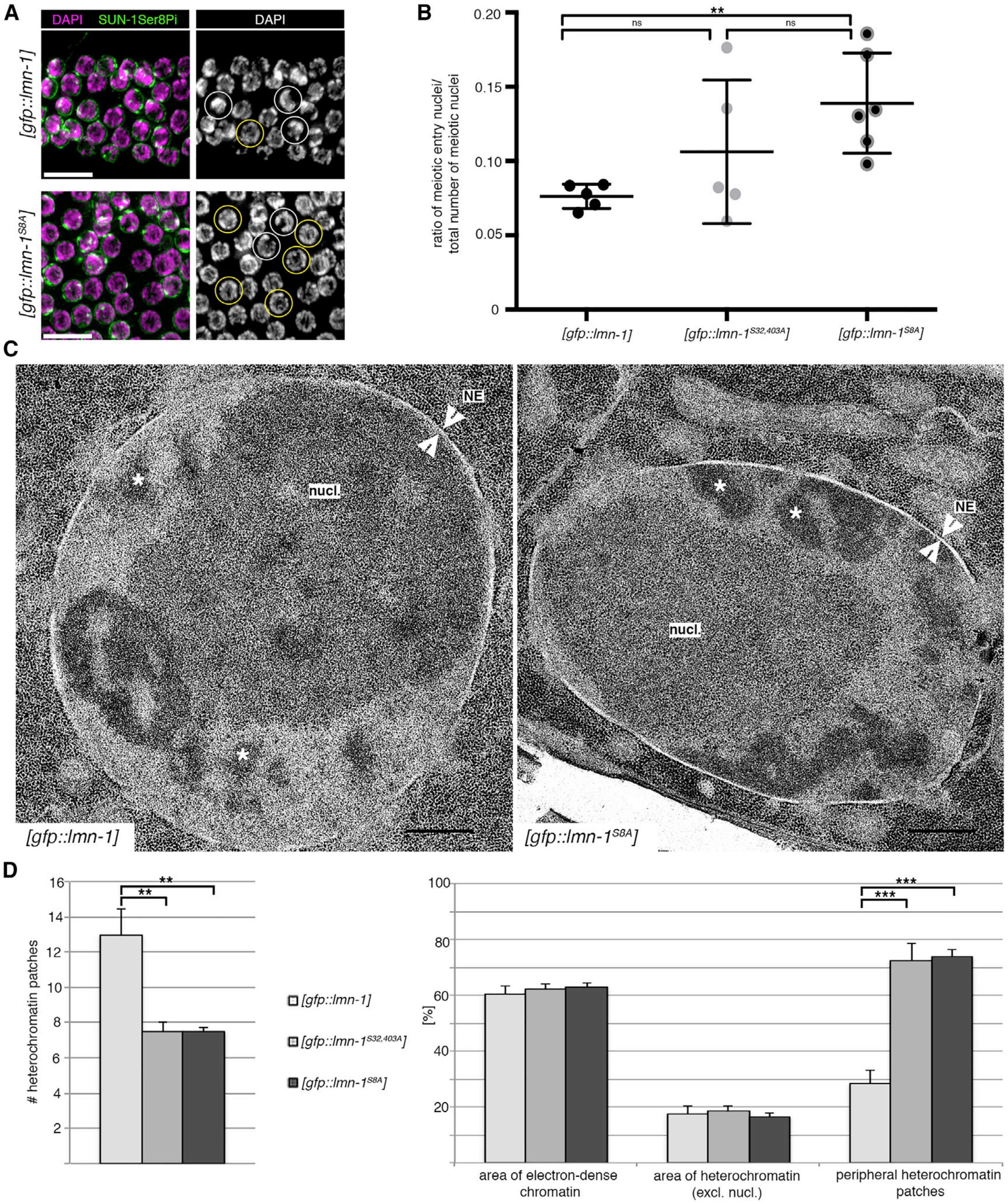

Figure 4. Mutants Expressing Non-phosphorylatable LMN-1 Have Delayed Leptotene Progression and Altered Heterochromatin Distribution in Transition Zone/Early Pachytene Nuclei

(A) Nuclei at meiotic entry have positive SUN-1Ser8pi staining and a non-polarized/spherical DAPI-staining pattern (yellow circles). Zygotene nuclei are also positive for SUN-1Ser8pi staining but have a polarized/crescent DAPI-staining pattern (white circles). Scale bars, 10 μm.

(B) Nuclei positive for SUN-1Ser8pi at meiotic entry in the wild-type and mutants expressing non-phosphorylatable LMN-1 were counted in all rows from meiotic entry until full SC polymerization, and values were normalized to the total number of nuclei from meiotic entry to diplotene. [*gfp::lmn-1*], n = 5 gonads; [*gfp::lmn-1^{S32,403A}*], n = 5 gonads; [*gfp::lmn-1^{S8A}*], n = 6 gonads. ns, not significant ($p > 0.05$); **$p = 0.0043$. p values were calculated using the Mann-Whitney U test. Scatter plots indicate the mean and SD. For additional classification of meiotic entry nuclei, see Figure S4.

(C) Representative electron micrographs of early meiotic nuclei from the wild-type and mutants expressing non-phosphorylatable LMN-1. Regions of higher electron density within the nucleus represent heterochromatin (star) and the large, polarized nucleolus (nucl.); arrowheads indicate the position of the nuclear envelope (NE). Scale bars, 0.5 μm.

(D) Number and distribution of heterochromatin patches in representative electron micrographs of nuclei from wild-type and mutants expressing non-phosphorylatable LMN-1. See Table S2 for further information on the choice of nuclei. [*gfp::lmn-1*], n = 9 nuclei; [*gfp::lmn-1^{S32,403A}*], n = 8 nuclei; [*gfp::lmn-1^{S8A}*], n = 8

(legend continued on next page)

SC formation can be monitored by the degree of colocalization of axial and central element components (MacQueen et al., 2002). X chromosome pairing is monitored by staining for the PC protein HIM-8 (Phillips et al., 2005). Although the timing of RAD-51 appearance and disappearance in both *lmn-1* mutants was comparable with that of the wild-type, excess RAD-51 accumulated in [*gfp*::*lmn-1*^{S8A}] germlines at the later stages of prophase, indicating an accumulation of recombination intermediates (Figures 5B and S5A). Additionally, [*gfp*::*lmn-1*^{S8A}] germlines had a similar proportion of nuclei with unpaired HIM-8 signals at earlier stages as well as a slight overall delay in X chromosome pairing (Figures 5C and S5B). The length of the SUN-1Ser8pi-positive zone was more variable in [*gfp*::*lmn-1*^{S8A}] than in wild-type gonads (Figure 5D). Nevertheless, the kinetics of SC formation was comparable (Figure S5C). Altogether, these findings indicate that recombination intermediates may be processed aberrantly and chromosome pairing was impeded in some nuclei.

Since these defects did not lead to a lower viability in our mutants, we hypothesized that nuclei with aberrant pairing might be removed via apoptosis. Defective meiotic cells are usually culled by programmed apoptosis in pachytene (Gartner et al., 2000). Indeed, the two non-phosphorylatable *lmn-1* mutants (particularly [*gfp*::*lmn-1*^{S8A}]) showed elevated germline apoptosis (Figures 5E and S5D), consistent with an increased frequency of meiotic errors. Preventing germline apoptosis by introducing the *ced-3*(*n717*) mutation enables the analysis of all diakinesis chromosomes, which otherwise might be culled. Chromosomal aberrations were rare in diakinesis oocytes expressing non-phosphorylatable LMN-1 in the apoptosis-proficient background, but more frequent in the *ced-3* mutant (Figures 5F and 5G). Fused chromosome structures were more common in both *lmn-1* mutant strains in the absence of germline apoptosis. The significantly decreased viability of the *lmn-1* mutants deficient for apoptosis further demonstrates that a substantial amount of germ cells generated in the presence of non-phosphorylatable *lmn-1* contains chromosomal defects incompatible with offspring viability (hatch rate in wild-type 95.4%, versus 74.3% in the mutant; see Table S1 for brood sizes and viabilities). Combining [*gfp*::*lmn-1*] and [*gfp*::*lmn-1*^{S8A}] with *spo-11*, to prevent meiotic DSB induction, led to the formation of 12 univalents, which excludes that the observed defects were caused by mitotic errors or aneuploidies (Figure 5G).

Coexpression of Non-phosphorylatable LMN-1 and SUN-1 Results in a Synthetic Phenotype with Aberrant Chromosome Configurations

We previously showed that phospho-modified SUN-1 forms part of a feedback mechanism that extends prophase I when meiotic tasks are unfinished (Woglar et al., 2013). Findings of RAD-51 accumulation in some nuclei, increased apoptosis, and variable duration of phospho-SUN-1 modification in the non-phosphorylatable lamin mutant prompted us to investigate the [*gfp*::*lmn-1*^{S8A}] mutation in a background that prevents extension of the movement process, [*sun-1*^{S10A}::*gfp*] (for strain viability, see Table S1). Diakinesis oocytes in single mutants or control strains had six DAPI-stained bodies in >90% of diakinesis nuclei (Figures 5F, 5G, 6A, and 6B). In double mutants expressing either partial or complete non-phosphorylatable LMN-1 alleles together with non-phosphorylatable SUN-1, the proportion of diakinesis nuclei containing six DAPI-stained bodies was significantly reduced to 71.7% and 64.9%, respectively. Strikingly, in the [*gfp*::*lmn-1*^{S8A}];[*sun-1*^{S10A}::*gfp*] mutant, approximately 30% of diakinesis nuclei contained fused chromosome structures. Fluorescent *in situ* hybridization (FISH) analysis of [*gfp*::*lmn-1*^{S8A}];[*sun-1*^{S10A}::*gfp*] showed that a similar proportion of nuclei within the last 10 rows of the pachytene stage showed unpaired FISH signals (Figure 6C), indicating a pairing/synapsis defect (71 nuclei with unpaired FISH signal of 283 analyzed nuclei corresponding to 25.1% with unpaired FISH signals in [*gfp*::*lmn-1*^{S8A}];[*sun-1*^{S10A}::*gfp*] versus 20 nuclei with unpaired FISH signal of 289 analyzed nuclei, corresponding to 6.9% in [*gfp*::*lmn-1*];[*sun-1*::*gfp*], chi-square $p < 0.0001$, versus 50 nuclei with unpaired FISH signal of 299 analyzed nuclei, corresponding to 16.8% in the [*gfp*::*lmn-1*];[*sun-1*^{S10A}::*gfp*], chi-square $p < 0.0129$). Furthermore, combining the non-phosphorylatable double mutant with the apoptosis-deficient *ced-3*(*n717*) mutation revealed the full magnitude of the synthetic phenotype. In [*gfp*::*lmn-1*^{S8A}];[*sun-1*^{S10A}::*gfp*] *ced-3* mutants, only 30% of all analyzed diakinesis showed six intact bivalents (Figure 6B′). Even though a portion of the observed univalents seem to stem from the [*sun-1*^{S10A}::*gfp*] mutation alone, the synthetic phenotype in the absence of germline apoptosis is significantly exacerbated. Aberrant chromatin masses and fused chromosomes were absent in the DSB-deficient *spo-11* mutant; therefore, aberrant joined chromosome structures must depend on DNA DSB induction and/or recombination (Figure 6B′).

Notably, in the [*gfp*::*lmn-1*^{S8A}];[*sun-1*^{S10A}::*gfp*] mutant, large unstructured chromatin masses were present in diakinesis (Figure 6A). To further investigate the origin of these chromosomal fusions, we performed super-resolution imaging of chromosome spreads stained for components of axial and central SC elements (Figure 6D). Pachytene nuclei contained chromosomal interlocks only in those double mutants expressing both non-phosphorylatable LMN-1 and non-phosphorylatable SUN-1. These interlocks occurred in late pachytene nuclei (as judged by the amount of synapsis), at a frequency of 17% in the [*gfp*::*lmn-1*^{S32,403A}];[*sun-1*::*gfp*^{S10A}] mutant and 20% in the [*gfp*::*lmn-1*^{S8A}];[*sun-1*^{S10A}::*gfp*] mutant (n = 94 and n = 64 pachytene nuclei, respectively). More complex chromosomal aberrations were also observed, but their origin was more difficult to deduce. Interlocks were never observed in pachytene nuclei of control genotypes or in any single mutant (n = 56 pachytene nuclei in [*gfp*::*lmn-1*];[*sun-1*::*gfp*], n = 48 pachytene nuclei in [*sun-1*^{S10A}::*gfp*], and n = 36 pachytene nuclei in [*gfp*::*lmn-1*^{S8A}]).

These synthetic phenotypes indicate that the unfavorable chromosome configurations seen in mutants expressing non-phosphorylatable lamin alleles are eliminated by extension of the movement process or that LMN-1 and SUN-1 phosphorylation

nuclei. For the number of heterochromatin patches, **$p = 0.005$ for [*gfp*::*lmn-1*] versus [*gfp*::*lmn-1*^{S32,403A}], **$p = 0.004$ for [*gfp*::*lmn-1*] versus [*gfp*::*lmn-1*^{S8A}], and $p > 0.05$ (ns) is not indicated; for the percentage area of heterochromatin and peripheral heterochromatin, ***$p = 8.9 \times 10^{-5}$ for [*gfp*::*lmn-1*] versus [*gfp*::*lmn-1*^{S32,304A}], ***$p = 2.5 \times 10^{-6}$ for [*gfp*::*lmn-1*] versus [*gfp*::*lmn-1*^{S8A}], and $p > 0.05$ (ns) not indicated for all others. p values were calculated using the two-tailed Student's t test. Error bars represent SD.

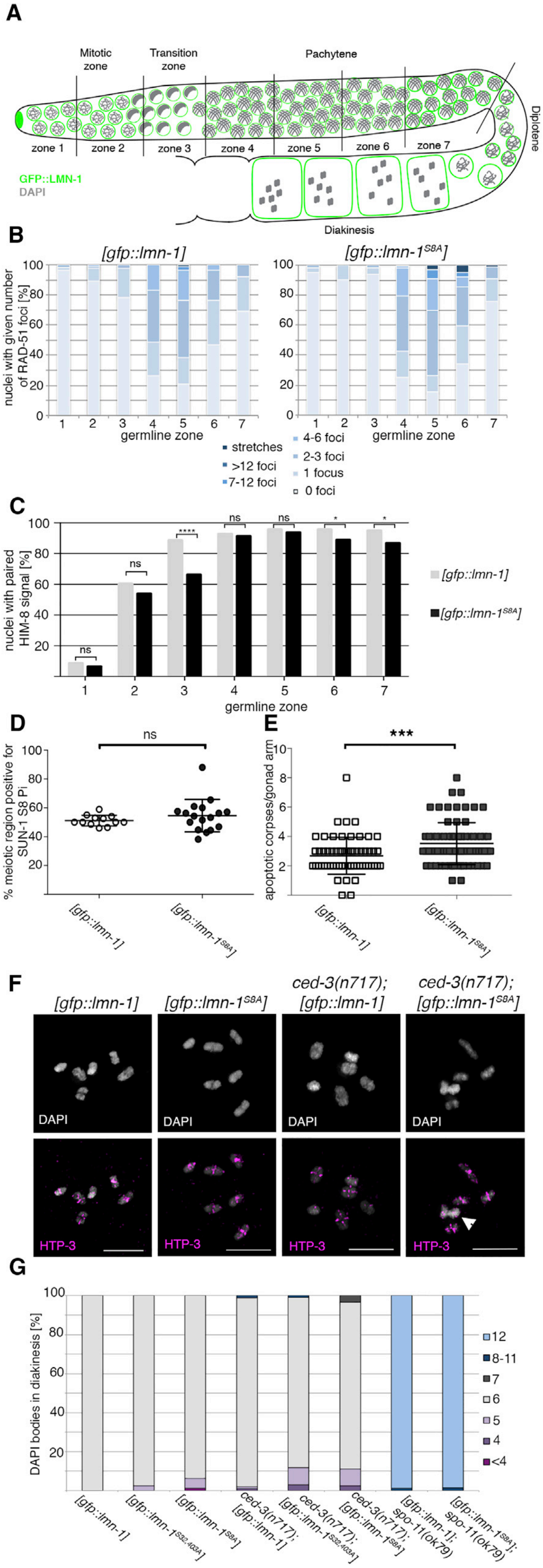

Figure 5. Mutants Expressing Non-phosphorylatable LMN-1 Display Aberrant Recombination Intermediates and Elevated Germline Apoptosis, Concomitant with an Occasional Delay in Meiotic Progression and Pairing

(A) Schematic representation of a *C. elegans* gonad, showing all meiotic stages in spatial and temporal order. Gonads were divided into seven zones of equal size for describing the behavior of markers along the time course of prophase I. See Figure S5 for additional data.

(B) Percentage of nuclei with a given number of RAD-51 foci (indicative of meiotic DNA DSB induction and repair kinetics) in the wild-type and non-phosphorylatable *lmn-1* mutant. [*gfp*::*lmn-1*], n = 3 gonads; [*gfp*::*lmn-1*^{S8A}], n = 3 gonads.

(C) X chromosome pairing kinetics, as indicated by percentages of nuclei containing paired or unpaired signals for the X chromosomal pairing center protein, HIM-8, in wild-type and [*gfp*::*lmn-1* ^{S8A}] gonads. [*gfp*::*lmn-1*], n = 5 gonads; [*gfp*::*lmn-1*^{S8A}], n = 4 gonads. p values were calculated using the chi-square test: p = 0.5726 (ns), 0.3149 (ns), ****p < 0.001, p = 0.6538 (ns), p = 0.4578 (ns), *p = 0.0448, and *p = 0.0247 for each germline zone, respectively.

(D) Percentage of the meiotic region with SUN-1Ser8pi staining, indicating ongoing recombination and SC formation. [*gfp*::*lmn-1*], n = 12 gonads; [*gfp*::*lmn-1*^{S8A}], n = 17 gonads. p = 0.3179 (ns). Scatter plots indicate mean and SD.

(E) Quantification of SYTO-12-positive apoptotic corpses per gonad arm. [*gfp*::*lmn-1*], n = 61 gonads; [*gfp*::*lmn-1*^{S8A}], n = 77 gonads. ***p = 0.0002, calculated using the Mann-Whitney U test. Scatter plots indicate mean and SD.

(F) Representative images of diakinesis oocytes expressing either wild-type or non-phosphorylatable LMN-1 in an apoptosis-proficient background and in the *ced-3*(*n717*) apoptosis mutant. The arrowhead indicates aberrantly joined DAPI-positive structures. Scale bars, 5 μm.

(G) Quantification of DAPI-stained bodies in -1 and -2 diakinesis oocytes in the presence or absence of germline apoptosis (*ced-3*) and deficient for DNA DSBs (*spo-11*(*ok79*)). [*gfp*::*lmn-1*], n = 50 oocytes; [*gfp*::*lmn-1*^{S32,403A}], n = 83 oocytes; [*gfp*::*lmn-1*^{S8A}], n = 78 oocytes. In the *ced-3* mutant background: [*gfp*::*lmn-1*], n = 94 oocytes; [*gfp*::*lmn-1*^{S32,403A}], n = 101 oocytes; [*gfp*::*lmn-1*^{S8A}], n = 118 oocytes. In the *spo-11* mutant background: [*gfp*::*lmn-1*], n = 58 oocytes; [*gfp*::*lmn-1*^{S8A}], n = 63 oocytes.

contribute to redundant processes in meiotic chromosome reorganization, or both. Chromosome aberrations result from stochastic events and therefore do not occur in all nuclei.

DISCUSSION

We propose that during the period of vigorous meiotic chromosome movement the nuclear lamina undergoes structural changes similar to those seen during nuclear envelope breakdown, without being fully eliminated. Phosphorylation of clustered residues flanking the LMN-1 coiled-coil domain occurs after meiotic entry, coincident with the formation of a more "soluble" lamina. Previous biochemical experiments showed that similar modifications lead to the disassembly of head-to-tail lamin dimers, resulting in a reversal of lamina crosslinks (Peter et al., 1991). In prophase of meiosis I, weakening of the crosslinks leads to a marked decrease in the amount of lamin at the nuclear periphery.

Until now, the extent to which the rigidity of the lamina itself impedes chromosome mobility was unclear. Surprisingly, we found that tubulin-driven cytoskeletal forces can overcome the constraint of a more "rigid" lamina: most offspring, even from the strongest [*gfp*::*lmn-1*^{S8A}] allele, are viable and chromosomes engage in productive movement. Note that the complete abrogation of movement results in univalents at diakinesis and highly

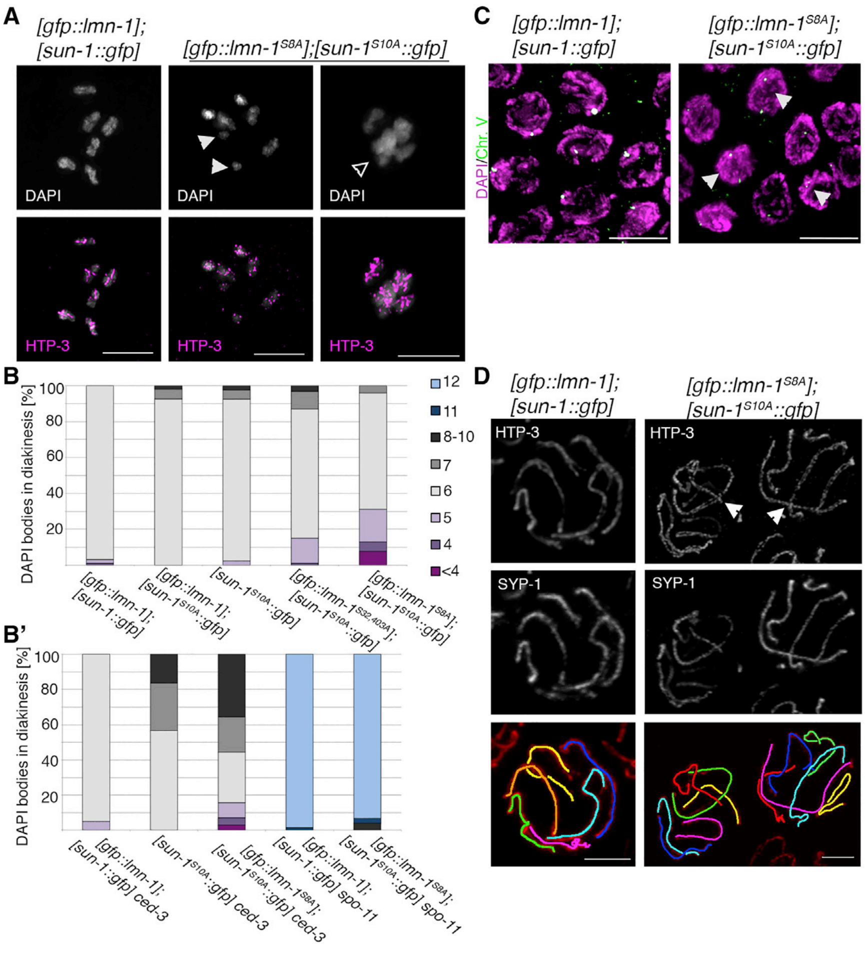

Figure 6. Worms Expressing Non-phosphorylatable LMN-1 and SUN-1 Proteins Have a Synthetic Meiotic Phenotype Consisting of Chromosome Interlocks and Aberrant Chromatin Configurations at Diakinesis

(A) Representative images of diakinesis chromosomes from the wild-type and the [*gfp*::*lmn-1*^{S8A}]; [*sun-1*^{S10A}::*gfp*] double mutant expressing non-phosphorylatable LMN-1 and SUN-1, stained with DAPI and HTP-3. Filled arrowheads indicate smaller non-bivalent DAPI structures (univalents or fragments); the open arrowhead indicates fused chromatin masses. Scale bars, 5 μm.

(B and B′) Quantification of DAPI-stained bodies in -1 and -2 oocytes from control and [*gfp*::*lmn-1*^{S8A}];[*sun-1*^{S10A}::*gfp*] mutants. [*gfp*::*lmn-1*]; [*sun-1*::*gfp*], n = 93 oocytes; [*gfp*::*lmn-1*];[*sun-1*^{S10A}::*gfp*], n = 54 oocytes; [*sun-1*^{S10A}::*gfp*], n = 79 oocytes; [*gfp*::*lmn-1*^{S32,403A}];[*sun-1*^{S10A}::*gfp*], n = 92 oocytes; [*gfp*::*lmn-1*^{S8A}];[*sun-1*^{S10A}::*gfp*], n = 77 oocytes; [*gfp*::*lmn-1*];[*sun-1*::*gfp*] *ced-3*, n = 70 oocytes; [*sun-1*^{S10A}::*gfp*] *ced-3*, n = 67 oocytes; [*gfp*::*lmn-1*^{S8A}];[*sun-1*^{S10A}::*gfp*] *ced-3*, n = 81 oocytes; [*gfp*::*lmn-1*];[*sun-1*::*gfp*] *spo-11*(*ok79*), n = 73 oocytes; [*gfp*::*lmn-1*^{S8A}]; [*sun-1*^{S10A}::*gfp*] *spo-11*(*ok79*), n = 74 oocytes.

(C) FISH analysis of the wild-type and [*gfp*::*lmn-1*^{S8A}]; [*sun-1*^{S10A}::*gfp*] using a probe for the 5S rDNA locus (chromosome V). Images show a region within the last 10 rows of pachytene nuclei. Arrowheads indicate unpaired FISH signals. Scale bars, 5 μm.

(D) Upper panels: super-resolution Airyscan images showing the localization of axial (HTP-3) and central (SYP-1) SC components in chromosome spreads of control and [*gfp*::*lmn-1*^{S8A}]; [*sun-1*^{S10A}::*gfp*] mutant pachytene nuclei. Half nuclei are projected for these images. Lower panel: false color labeling of the six paired homologous chromosomes for better visualization. Arrowheads indicate chromosomal interlocks in nuclei with complete synapsis. Scale bar, 3 μm.

reduced fertility (Penkner et al., 2007). The defects we observed, mostly aberrantly joined chromosome structures (interlocks are easiest to interpret; however, unstructured chromatin masses have also been observed), unpaired homologs, and unscheduled RAD-51 accumulation, occur stochastically and at low frequency. Those defects can best be explained by defective chromatin reorganization and/or removal from the nuclear periphery. It is likely that extensive chromatin removal is necessary to prevent chromosomes that are being dragged through the nuclear envelope from becoming entangled with other chromosomes with ectopic connections to the nuclear periphery. Indeed, the accumulation of nuclei displaying features of meiotic entry/leptotene in [*gfp*::*lmn-1*^{S8A}] strongly supports the hypothesis that chromatin reorganization is compromised. Mammals might have devised the opening of the network by implementation of the shortened more mobile lamin C2 isoform (Jahn et al., 2010; Link et al., 2013); however, opening of the lamin B network is still unresolved.

The lamina is a highly interconnected three-dimensional network, in which lamin-chromatin connections occur at many different levels—for example, lamin-LADs (lamina-associated domains of chromatin), lamin-pores (with pores themselves connected to chromatin), lamin-LEM-domain proteins, which can be connected to chromatin via BAF1, to name just a few (Yáñez-Cuna and van Steensel, 2017). In interphase nuclei, it has already been demonstrated that nuclear pores and lamin B form tightly connected networks (Daigle et al., 2001). We propose that during meiosis lamina downsizing decreases the number of chromatin-nuclear envelope connections, thus promoting chromatin reorganization. Alternatively, the meiotic lamina could be more prone to turnover, thus promoting less stable interactions within the three-dimensional network. FRAP (fluorescence recovery after photobleaching) experiments, however, did not show significant recovery after photobleaching in mitotic or meiotic nuclei in both [*gfp*::*lmn-1*] and [*gfp*::*lmn-1*^{S8A}] worms (Figure S6). This argues against an accelerated exchange of lamin proteins.

The modest reduction in chromosome end velocity could explain the delayed pairing, but has no significant downstream consequences for global synapsis. The existence of efficient mechanisms to resolve topologically inappropriate relationships among chromosomes during alignment was previously reported

in *Sordaria*. Here, interlocked chromosomes are observed in 20% of wild-type zygotene nuclei, but have disappeared by pachytene (Storlazzi et al., 2010). The "self-correction" of inappropriate chromosome configurations is also accomplished through activation of a surveillance system, resulting in extension of the chromosome movement process mediated by SUN-1 phosphorylation (Woglar et al., 2013). Besides the culling of affected nuclei by apoptosis (as we observed in [*gfp*::*lmn-1*^{S8A}] gonads), activation of these self-correction mechanisms in the parent could explain the high viability of mutant offspring.

This study provides a framework for understanding how the nuclear lamina is modified during chromosome movement in meiotic prophase, and which chromosome anomalies we can expect if chromatin reorganization is impaired or delayed.

STAR★METHODS

Detailed methods are provided in the online version of this paper and include the following:

- KEY RESOURCES TABLE
- CONTACT FOR REAGENT AND RESOURCE SHARING
- EXPERIMENTAL MODEL AND SUBJECT DETAILS
- METHOD DETAILS
  - Detergent Extraction Assay
  - Immunofluorescence Analysis
  - Chromosome Spreads
  - FISH Analysis
  - SYTO-12 Staining
  - Image Acquisition
  - Live Imaging of Worms for Monitoring of Chromosome Movement
  - Live Imaging of Worms for Monitoring of the First Embryonic Division
  - FRAP Analysis of the Nuclear Lamina in Living Worms
  - Electron Microscopy
  - Proteomic Analysis
- QUANTIFICATION AND STATISTICAL ANALYSIS
  - Quantification of RAD-51 Foci
  - Quantification of X-Chromosome Pairing
  - Quantification of SC Assembly
  - Quantification of Fluorescence Signal Intensities
  - Quantification of FRAP Signal Intensities
  - Raw Data Processing of Proteomic MS Data
  - Statistical Analysis

SUPPLEMENTAL INFORMATION

Supplemental Information includes six figures and three tables and can be found with this article online at https://doi.org/10.1016/j.devcel.2018.03.018.

ACKNOWLEDGMENTS

We thank the Caenorhabditis Genetics Center (funded by NIH Office of Research Infrastructure Programs P40 OD010440) for worm strains; Georg Krohne, Sarit Smolikove, Abby Dernburg, and Anne Villeneuve for antibodies; and Angela Graf and Silke Braune for technical help. We are indebted to Roland Foisner, Pawel Pasierbek, Christian Frøkjaer-Jensen, Josef Gotzmann, Thomas Peterbauer, and members of the Jantsch and Loidl laboratories for helpful discussions and advice. The Jantsch laboratory is supported by the Austrian Science Fund (FWF; project no. SFB F3415-B19). D.P. and T.L. were supported by the Austrian Science Fund doctoral program (project no. W1238). J.L. was awarded an EMBO Long-Term Fellowship (EMBO ALTF 403-2014). S.M.M. was supported by a PhD grant from the Studienstiftung des Deutschen Volkes. The A.D. laboratory was funded by a START grant project (no. Y597-B20) from the Austrian Science Fund. E.M.-P. is funded by the Medical Research Council (grant no. MC-A652–5PY60). M.Z. acknowledges funds from the Canadian Institutes of Health Research.

AUTHOR CONTRIBUTIONS

D.P. and J.L. conducted and analyzed all cell biology experiments; M.V., A.D., D.P., and J.L. constructed the worm strains; C.S. and M.A. performed the electron microscopy analysis, together with J.L. and S.M.M.; A.M., C.B., E.M.-P., H.K., A.W., and S.P.-P. generated liquid chromatography-tandem mass spectrometry data; A.D. and T.L. performed *in vivo* imaging and analyzed embryonic divisions; and M.Z. and S.L. generated the *plk-2* mutant strain. A.B. contributed the graphical abstract. D.P., J.L., and V.J. conceived the project, analyzed data, and wrote the manuscript.

DECLARATION OF INTERESTS

The authors declare no competing interests.

Received: October 10, 2017
Revised: February 13, 2018
Accepted: March 23, 2018
Published: April 23, 2018

REFERENCES

Arribere, J.A., Bell, R.T., Fu, B.X., Artiles, K.L., Hartman, P.S., and Fire, A.Z. (2014). Efficient marker-free recovery of custom genetic modifications with CRISPR/Cas9 in *Caenorhabditis elegans*. Genetics *198*, 837–846.

Baudrimont, A., Penkner, A., Woglar, A., Machacek, T., Wegrostek, C., Gloggnitzer, J., Fridkin, A., Klein, F., Gruenbaum, Y., Pasierbek, P., and Jantsch, V. (2010). Leptotene/zygotene chromosome movement via the SUN/KASH protein bridge in *Caenorhabditis elegans*. PLoS Genet. *6*, e1001219.

Brenner, S. (1974). The genetics of *Caenorhabditis elegans*. Genetics *77*, 71–94.

Casado, P., Bilanges, B., Rajeeve, V., Vanhaesebroeck, B., and Cutillas, P.R. (2014). Environmental stress affects the activity of metabolic and growth factor signaling networks and induces autophagy markers in MCF7 breast cancer cells. Mol. Cell. Proteomics *13*, 836–848.

Cohen-Fix, O., and Askjaer, P. (2017). Cell biology of the *Caenorhabditis elegans* nucleus. Genetics *205*, 25–59.

Colaiacovo, M.P., MacQueen, A.J., Martinez-Perez, E., McDonald, K., Adamo, A., La Volpe, A., and Villeneuve, A.M. (2003). Synaptonemal complex assembly in *C. elegans* is dispensable for loading strand-exchange proteins but critical for proper completion of recombination. Dev. Cell *5*, 463–474.

Daigle, N., Beaudouin, J., Hartnell, L., Imreh, G., Hallberg, E., Lippincott-Schwartz, J., and Ellenberg, J. (2001). Nuclear pore complexes form immobile networks and have a very low turnover in live mammalian cells. J. Cell Biol. *154*, 71–84.

Dechat, T., Adam, S.A., and Goldman, R.D. (2009). Nuclear lamins and chromatin: when structure meets function. Adv. Enzyme Regul. *49*, 157–166.

Dernburg, A.F., McDonald, K., Moulder, G., Barstead, R., Dresser, M., and Villeneuve, A.M. (1998). Meiotic recombination in *C. elegans* initiates by a conserved mechanism and is dispensable for homologous chromosome synapsis. Cell *94*, 387–398.

Foeger, N., Wiesel, N., Lotsch, D., Mücke, N., Kreplak, L., Aebi, U., Gruenbaum, Y., and Herrmann, H. (2006). Solubility properties and specific assembly pathways of the B-type lamin from *Caenorhabditis elegans*. J. Struct. Biol. *155*, 340–350.

Friedland, A.E., Tzur, Y.B., Esvelt, K.M., Colaiácovo, M.P., Church, G.M., and Calarco, J.A. (2013). Heritable genome editing in *C. elegans* via a CRISPR-Cas9 system. Nat. Methods *10*, 741–743.

Frøkjaer-Jensen, C., Davis, M.W., Hopkins, C.E., Newman, B.J., Thummel, J.M., Olesen, S.P., Grunnet, M., and Jorgensen, E.M. (2008). Single-copy insertion of transgenes in *Caenorhabditis elegans*. Nat. Genet. *40*, 1375–1383.

Gartner, A., Milstein, S., Ahmed, S., Hodgkin, J., and Hengartner, M.O. (2000). A conserved checkpoint pathway mediates DNA damage-induced apoptosis and cell cycle arrest in *C. elegans*. Mol. Cell *5*, 435–443.

Gerace, L., and Blobel, G. (1980). The nuclear envelope lamina is reversibly depolymerized during mitosis. Cell *19*, 277–287.

Goodyer, W., Kaitna, S., Couteau, F., Ward, J.D., Boulton, S.J., and Zetka, M. (2008). HTP-3 links DSB formation with homolog pairing and crossing over during *C. elegans* meiosis. Dev. Cell *14*, 263–274.

Güttinger, S., Laurell, E., and Kutay, U. (2009). Orchestrating nuclear envelope disassembly and reassembly during mitosis. Nat. Rev. Mol. Cell Biol. *10*, 178–191.

Haithcock, E., Dayani, Y., Neufeld, E., Zahand, A.J., Feinstein, N., Mattout, A., Gruenbaum, Y., and Liu, J. (2005). Age-related changes of nuclear architecture in *Caenorhabditis elegans*. Proc. Natl. Acad. Sci. USA *102*, 16690–16695.

Harper, N.C., Rillo, R., Jover-Gil, S., Assaf, Z.J., Bhalla, N., and Dernburg, A.F. (2011). Pairing centers recruit a Polo-like kinase to orchestrate meiotic chromosome dynamics in *C. elegans*. Dev. Cell *21*, 934–947.

Heald, R., and McKeon, F. (1990). Mutations of phosphorylation sites in lamin A that prevent nuclear lamina disassembly in mitosis. Cell *61*, 579–589.

Heitlinger, E., Peter, M., Häner, M., Lustig, A., Aebi, U., and Nigg, E.A. (1991). Expression of chicken lamin B2 in *Escherichia coli*: characterization of its structure, assembly, and molecular interactions. J. Cell Biol. *113*, 485–495.

Hillers, K.J., Jantsch, V., Martinez-Perez, E., and Yanowitz, J.L. (2017). Meiosis (WormBook), pp. 1–43.

Hiraoka, Y., and Dernburg, A.F. (2009). The SUN rises on meiotic chromosome dynamics. Dev. Cell *17*, 598–605.

Jahn, D., Schramm, S., Benavente, R., and Alsheimer, M. (2010). Dynamic properties of meiosis-specific lamin C2 and its impact on nuclear envelope integrity. Nucleus *1*, 273–283.

Jantsch, V., Tang, L., Pasierbek, P., Penkner, A., Nayak, S., Baudrimont, A., Schedl, T., Gartner, A., and Loidl, J. (2007). *Caenorhabditis elegans* prom-1 is required for meiotic prophase progression and homologous chromosome pairing. Mol. Biol. Cell *18*, 4911–4920.

Karabinos, A., Schunemann, J., Meyer, M., Aebi, U., and Weber, K. (2003). The single nuclear lamin of *Caenorhabditis elegans* forms in vitro stable intermediate filaments and paracrystals with a reduced axial periodicity. J. Mol. Biol. *325*, 241–247.

Kim, Y., Kostow, N., and Dernburg, A.F. (2015). The chromosome axis mediates feedback control of CHK-2 to ensure crossover formation in *C. elegans*. Dev. Cell *35*, 247–261.

Koszul, R., and Kleckner, N. (2009). Dynamic chromosome movements during meiosis: a way to eliminate unwanted connections? Trends Cell Biol. *19*, 716–724.

Kutay, U., and Hetzer, M.W. (2008). Reorganization of the nuclear envelope during open mitosis. Curr. Opin. Cell Biol. *20*, 669–677.

Labella, S., Woglar, A., Jantsch, V., and Zetka, M. (2011). Polo kinases establish links between meiotic chromosomes and cytoskeletal forces essential for homolog pairing. Dev. Cell *21*, 948–958.

Link, J., Jahn, D., Schmitt, J., Göb, E., Baar, J., Ortega, S., Benavente, R., and Alsheimer, M. (2013). The meiotic nuclear lamina regulates chromosome dynamics and promotes efficient homologous recombination in the mouse. PLoS Genet. *9*, e1003261.

Liu, J., Rolef Ben-Shahar, T., Riemer, D., Treinin, M., Spann, P., Weber, K., Fire, A., and Gruenbaum, Y. (2000). Essential roles for *Caenorhabditis elegans* lamin gene in nuclear organization, cell cycle progression, and spatial organization of nuclear pore complexes. Mol. Biol. Cell *11*, 3937–3947.

MacQueen, A.J., Colaiacovo, M.P., McDonald, K., and Villeneuve, A.M. (2002). Synapsis-dependent and -independent mechanisms stabilize homolog pairing during meiotic prophase in *C. elegans*. Genes Dev. *16*, 2428–2442.

Martinez-Perez, E., and Villeneuve, A.M. (2005). HTP-1-dependent constraints coordinate homolog pairing and synapsis and promote chiasma formation during *C. elegans* meiosis. Genes Dev. *19*, 2727–2743.

Mattout, A., Pike, B.L., Towbin, B.D., Bank, E.M., Gonzalez-Sandoval, A., Stadler, M.B., Meister, P., Gruenbaum, Y., and Gasser, S.M. (2011). An EDMD mutation in *C. elegans* lamin blocks muscle-specific gene relocation and compromises muscle integrity. Curr. Biol. *21*, 1603–1614.

Mlynarczyk-Evans, S., and Villeneuve, A.M. (2017). Time-course analysis of early meiotic prophase events informs mechanisms of homolog pairing and synapsis in *Caenorhabditis elegans*. Genetics *207*, 103–114.

Monen, J., Maddox, P.S., Hyndman, F., Oegema, K., and Desai, A. (2005). Differential role of CENP-A in the segregation of holocentric *C. elegans* chromosomes during meiosis and mitosis. Nat. Cell Biol. *7*, 1248–1255.

Naetar, N., Ferraioli, S., and Foisner, R. (2017). Lamins in the nuclear interior—life outside the lamina. J. Cell Sci. *130*, 2087–2096.

Pattabiraman, D., Roelens, B., Woglar, A., and Villeneuve, A.M. (2017). Meiotic recombination modulates the structure and dynamics of the synaptonemal complex during *C. elegans* meiosis. PLoS Genet. *13*, e1006670.

Penkner, A., Tang, L., Novatchkova, M., Ladurner, M., Fridkin, A., Gruenbaum, Y., Schweizer, D., Loidl, J., and Jantsch, V. (2007). The nuclear envelope protein Matefin/SUN-1 is required for homologous pairing in *C. elegans* meiosis. Dev. Cell *12*, 873–885.

Penkner, A.M., Fridkin, A., Gloggnitzer, J., Baudrimont, A., Machacek, T., Woglar, A., Csaszar, E., Pasierbek, P., Ammerer, G., Gruenbaum, Y., et al. (2009). Meiotic chromosome homology search involves modifications of the nuclear envelope protein Matefin/SUN-1. Cell *139*, 920–933.

Peter, M., Heitlinger, E., Häner, M., Aebi, U., and Nigg, E.A. (1991). Disassembly of in vitro formed lamin head-to-tail polymers by CDC2 kinase. EMBO J. *10*, 1535–1544.

Peter, M., Nakagawa, J., Dorée, M., Labbé, J.C., and Nigg, E.A. (1990). In vitro disassembly of the nuclear lamina and M phase-specific phosphorylation of lamins by cdc2 kinase. Cell *61*, 591–602.

Phillips, C.M., Wong, C., Bhalla, N., Carlton, P.M., Weiser, P., Meneely, P.M., and Dernburg, A.F. (2005). HIM-8 binds to the X chromosome pairing center and mediates chromosome-specific meiotic synapsis. Cell *123*, 1051–1063.

Rog, O., Köhler, S., and Dernburg, A.F. (2017). The synaptonemal complex has liquid crystalline properties and spatially regulates meiotic recombination factors. Elife *6*, https://doi.org/10.7554/eLife.21455.

Sato, A., Isaac, B., Phillips, C.M., Rillo, R., Carlton, P.M., Wynne, D.J., Kasad, R.A., and Dernburg, A.F. (2009). Cytoskeletal forces span the nuclear envelope to coordinate meiotic chromosome pairing and synapsis. Cell *139*, 907–919.

Silva, N., Ferrandiz, N., Barroso, C., Tognetti, S., Lightfoot, J., Telecan, O., Encheva, V., Faull, P., Hanni, S., Furger, A., et al. (2014). The fidelity of synaptonemal complex assembly is regulated by a signaling mechanism that controls early meiotic progression. Dev. Cell *31*, 503–511.

Simon, D.N., and Wilson, K.L. (2013). Partners and post-translational modifications of nuclear lamins. Chromosoma *122*, 13–31.

Stigloher, C., Zhan, H., Zhen, M., Richmond, J., and Bessereau, J.L. (2011). The presynaptic dense projection of the *Caenorhabditis elegans* cholinergic neuromuscular junction localizes synaptic vesicles at the active zone through SYD-2/liprin and UNC-10/RIM-dependent interactions. J. Neurosci. *31*, 4388–4396.

Storlazzi, A., Gargano, S., Ruprich-Robert, G., Falque, M., David, M., Kleckner, N., and Zickler, D. (2010). Recombination proteins mediate meiotic spatial chromosome organization and pairing. Cell *141*, 94–106.

CellPress

Stuurman, N., Heins, S., and Aebi, U. (1998). Nuclear lamins: their structure, assembly, and interactions. J. Struct. Biol. *122*, 42–66.

Torvaldson, E., Kochin, V., and Eriksson, J.E. (2015). Phosphorylation of lamins determine their structural properties and signaling functions. Nucleus *6*, 166–171.

Woglar, A., Daryabeigi, A., Adamo, A., Habacher, C., Machacek, T., La Volpe, A., and Jantsch, V. (2013). Matefin/SUN-1 phosphorylation is part of a surveillance mechanism to coordinate chromosome synapsis and recombination with meiotic progression and chromosome movement. PLoS Genet. *9*, e1003335.

Woglar, A., and Jantsch, V. (2014). Chromosome movement in meiosis I prophase of *Caenorhabditis elegans*. Chromosoma *123*, 15–24.

Wynne, D.J., Rog, O., Carlton, P.M., and Dernburg, A.F. (2012). Dynein-dependent processive chromosome motions promote homologous pairing in *C. elegans* meiosis. J. Cell Biol. *196*, 47–64.

Yáñez-Cuna, J.O., and van Steensel, B. (2017). Genome-nuclear lamina interactions: from cell populations to single cells. Curr. Opin. Genet. Dev. *43*, 67–72.

Zickler, D., and Kleckner, N. (1998). The leptotene-zygotene transition of meiosis. Annu. Rev. Genet. *32*, 619–697.

STAR★METHODS

KEY RESOURCES TABLE

REAGENT or RESOURCE	SOURCE	IDENTIFIER
**Antibodies**		
Anti-LMN-1	Gift from Georg Krohne, University of Würzburg, Germany	N/A
Anti-LMN-1Ser32pi	This paper	N/A
Anti-SUN-1Ser8pi	(Penkner et al., 2009)	N/A
Anti-mAB414	Abcam	Cat#ab5008
Anti-HTP-3	(Goodyer et al., 2008)	N/A
Anti-SYP-1	(MacQueen et al., 2002)	N/A
Anti-RAD-51	(Colaiacovo et al., 2003)	N/A
Anti-biotin	Abcam	Cat# ab6650; RRID: AB_305623
**Chemicals, Peptides, and Recombinant Proteins**		
SYTO-12	ThermoFischer Scientific	S7574
Vectashield Mounting Medium	Vector Labs	Cat#H-1000
(-)-Tetramisole hydrochloride	Sigma-Aldrich	Cat#L9756
**Critical Commercial Assays**		
Gateway BP Clonase II Enzyme Mix	ThermoFischer Scientific	Cat#11789020
Gateway LR Clonase II Enzyme Mix	ThermoFischer Scientific	Cat#11791100
DIG (Digoxigenin)-nick translation Kit	Sigma-Aldrich	Cat#11745816910
**Experimental Models: Organisms/Strains**		
C. elegans: N2 Bristol	CGC	https://cgc.umn.edu/strain/search
C. elegans: [*gfp::lmn-1*] *CrispR*: *lmn-1*(*jf98*[*gfp::lmn-1*]) *I*	This paper	UV117
C. elegans: [*gfp::lmn-1*]; *plk-2*(*K65M*): *plk-2*(*tm1395*) *lmn-1*(*jf98*[*gfp::lmn-1*]) *I*; *vvSi18* [*pie-1p::mCherry::plk-2*(*K65M*), *unc-119*(+)] *II*	This paper; plk-2(K65M) was obtained from Monique Zetka, McGill University, Canada	UV118
C. elegans: [*gfp::lmn-1*]; *chk-2*(*me64*): *lmn-1*(*jf98*[*gfp::lmn-1*]) *I*; *chk-2*(*me64*) *rol-9*(*sc148*)/*unc-51*(*e369*) *rol-9*(*sc148*) *V*	This paper	UV119
C. elegans: [*gfp::lmn-1*] *MosSCI*: *lmn-1*(*tm1502*) *I*; *jfSi68*[*Plmn-1::gfp cb-unc-119*(+)] *II*	This paper	UV120
C. elegans: [*gfp::lmn-1*^{S32,403A}] *MosSCI*: *lmn-1*(*tm1502*) *I*; *jfSi72*[*Plmn-1S*(*32, 403*)*A::gfp cb-unc-119*(+)] *II*	This paper	UV121
C. elegans: [*gfp::lmn-1*^{S8A}] *MosSCI*: *lmn-1*(*tm1502*) *I*; *jfSi89*[*Plmn-1S*(*21,22,24,32,397,398,403,405*)*A::gfp cb-unc-119*(+)] *II*	This paper	UV122
C. elegans: *ieDF2*: *ieDF2*/*mIs11*	(Harper et al., 2011)	ieDF2
C. elegans: *spo-11*(*ok79*): *spo-11*(*ok79*) *IV*/*nT1* [*qIs51*] (*IV*;*V*)	(Dernburg et al., 1998)	UV123
C. elegans: [*gfp::lmn-1*]; [*sun-1::mRuby*]: *lmn-1*(*tm1502*) *I*; *jfSi68*[*Plmn-1::gfp cb-unc-119*(+)] *II*; *ieSi21* [*sun-1p::sun-1::mRuby::sun-1 3′UTR* + *Cbr-unc-119*(+)] *IV*	This paper	UV124
C. elegans: [*gfp::lmn-1*^{S8A}]; [*sun-1::mRuby*]: *lmn-1*(*tm1502*) *I*; *jfSi89*[*Plmn-1S*(*21,22,24,32,397,398,403,405*)*A::gfp cb-unc-119*(+)] *II*; *ieSi21* [*sun-1p::sun-1::mRuby::sun-1 3′UTR* + *Cbr-unc-119*(+)] *IV*	This paper	UV125
C. elegans: [*gfp::lmn-1*]; [*him-8::mCherry*]; *sun-1*(*jf18*): *lmn-1*(*tm1502*) *I*; *jfSi68*[*Plmn-1::gfp cb-unc-119*(+)] *II*; *jfSi62* [*pie-1p::mCherry::him-8::unc-54 3′UTR cb-unc-119*(+)], *him-8*(*tm611*) *IV*; *sun-1*(*jf18*) *V*/*nT1* [*qIs51*] (*IV*;*V*)	This paper	UV126

(*Continued on next page*)

Continued

REAGENT or RESOURCE	SOURCE	IDENTIFIER
C. elegans: [*gfp::lmn-1*^{S32,403A}]; [*him-8::mCherry*]; *sun-1(jf18)*: *lmn-1(tm1502) I*; *jfSi72[Plmn-1S(32, 403)A::gfp cb-unc-119(+)] II*; *jfSi62 [pie-1p::mCherry::him-8::unc-54*3′*UTR cb-unc-119 (+)], him-8(tm611) IV; sun-1(jf18) V/nT1 [qIs51] (IV;V)*	This paper	UV127
C. elegans: [*gfp::lmn-1*^{S8A}]; [*him-8::mCherry*]; *sun-1(jf18)*: *lmn-1(tm1502) I*; *jfSi89[Plmn-1S(21, 22,24,32,397,398,403,405)A::gfp cb-unc-119(+)] II*; *jfSi62 [pie-1p::mCherry::him-8::unc-54*3′*UTR cb-unc-119 (+)], him-8(tm611) IV; sun-1(jf18) V/nT1 [qIs51] (IV;V)*	This paper	UV128
C. elegans: *ced-3*; [*gfp::lmn-1*]: *lmn-1(tm1502) I*; *jfSi68[Plmn-1::gfp cb-unc-119(+)] II*; *ced-3(n717) IV*	This paper	UV129
C. elegans: *ced-3*; [*gfp::lmn-1*^{S32,403A}]: *lmn-1(tm1502) I*; *jfSi72[Plmn-1S(32, 403)A::gfp cb-unc-119(+)] II*; *ced-3(n717) IV*	This paper	UV130
C. elegans: *ced-3*; [*gfp::lmn-1*^{S8A}]: *lmn-1(tm1502) I*; *jfSi89[Plmn-1S(21,22,24,32,397,398,403,405)A::gfp cb-unc-119(+)] II*; *ced-3(n717) IV*	This paper	UV131
C. elegans: [*gfp::lmn-1*]; *spo-11(ok79)*: *lmn-1(tm1502) I*; *jfSi68[Plmn-1::gfp cb-unc-119(+)] II*; *spo-11(ok79) IV/nT1 [qIs51] (IV;V)*	This paper	UV132
C. elegans: [*gfp::lmn-1*^{S8A}]; *spo-11(ok79)*: *lmn-1(tm1502) I*; *jfSi89[Plmn1S(21,22,24,32,397,398,403,405)A::gfp cb-unc-119(+)] II*; *spo-11(ok79) IV/nT1 [qIs51] (IV;V)*	This paper	UV133
C. elegans: [*sun-1*^{S10A}*::gfp*]*: *jfSi49[Psun-1::gfp S(8, 12, 16, 24, 36, 43, 58, 62, 69, 78)A cb-unc-119(+)] IV*; *sun-1 (ok1282) V*<br>*This strain contains the mutated phospho sites described in (Woglar et al., 2013) and three sites found with more sensitive mass spectrometry detection (16, 69, 78).	This paper	UV134
C. elegans: [*gfp::lmn-1*]; [*sun-1::gfp*]: *lmn-1(tm1502) I*; *jfSi68[Plmn-1::gfp cb-unc-119(+)] II*; *jfSi26[Psun-1::GFP cb-unc-119(+)] IV*; *sun-1(ok1282) V*	This paper	UV135
C. elegans: [*gfp::lmn-1*^{S32,403A}];[*sun-1*^{S10A}*::gfp*]: *lmn-1(tm1502) I*; *jfSi72[Plmn-1S(32, 403)A::gfp cb-unc-119(+)] II*; *jfSi49[Psun-1::gfp S(8, 12, 16, 24, 36, 43, 58, 62, 69, 78)A cb-unc-119(+)] IV*; *sun-1 (ok1282) V*	This paper	UV136
C. elegans: [*gfp::lmn-1*^{S8A}]; [*sun-1*^{S10A}*::gfp*]: *lmn-1(tm1502) I*; *jfSi89[Plmn-1S(21,22,24,32,397,398,403,405)A::gfp cb-unc-119(+)] II*; *jfSi49[Psun-1::gfp S(8, 12, 16, 24, 36, 43, 58, 62, 69, 78)A cb-unc-119(+)] IV*; *sun-1 (ok1282) V*	This paper	UV137
C. elegans: [*gfp::lmn-1*]; [*sun-1*^{S10A}*::gfp*]: *lmn-1(tm1502) I*; *jfSi68[Plmn-1::gfp cb-unc-119(+)] II*; *jfSi49[Psun-1::GFP S(8, 12, 16, 24, 36, 43, 58, 62, 69, 78)A cb-unc-119(+)] IV*; *sun-1 (ok1282) V*	This paper	UV138
C. elegans: [*lmn-1*^{S32,403,470A}]: *lmn-1(tm1502) I*; *jfSi76[Plmn-1S(32,403,470)A cb-unc-119(+)] II*	This paper	UV139
C. elegans: [*gfp::lmn-1*]; [*sun-1::gfp*] *spo-11(ok79)*: *lmn-1(tm1502) I*; *jfSi68[Plmn-1::gfp cb-unc-119(+)] II*; *jfSi26[Psun-1::gfp cb-unc-119(+)] spo-11(ok79) IV/nT1 [qIs51] (IV;V)*; *sun-1(ok1282) V/nT1 [qIs51] (IV;V)*	This paper	UV140

(*Continued on next page*)

Continued

REAGENT or RESOURCE	SOURCE	IDENTIFIER
C. elegans: [gfp::lmn-1^{S8A}]; [sun-1^{S10A}::gfp] spo-11(ok79): lmn-1(tm1502) I; jfSi89[Plmn-1S(21,22,24,32,397,398, 403,405)A::gfp cb-unc-119(+)] II; jfSi49[Psun-1::gfp S(8, 12, 16, 24, 36, 43, 58, 62, 69, 78)A cb-unc-119(+)] spo-11(ok79) IV/nT1 [qIs51] (IV;V); sun-1(ok1282) V/nT1 [qIs51] (IV;V)	This paper	UV141
C. elegans: [gfp::lmn-1]; histone::mCherry: lmn-1 (tm1502)I; jfSi68[Plmn-1::gfp cb-unc-119(+)] II; histone::mCherry IV	This paper	UV142
C. elegans: [gfp::lmn-1^{S32,403A}]; histone::mCherry: lmn-1(tm1502)I; jfSi72[Plmn-1S(32, 403)A::gfp cb-unc-119(+)] II; histone::mCherry IV	This paper	UV143
C. elegans: [gfp::lmn-1^{S8A}]; histone::mCherry: lmn-1 (tm1502)I; jfSi89[Plmn-1S(21,22,24,32,397,398,403, 405)A::gfp cb-unc-119(+)] II; histone::mCherry	This paper	UV144
C. elegans: [gfp::lmn-1]; [sun-1::gfp] ced-3(n717): lmn-1 (tm1502) I; jfSi68[Plmn-1::gfp cb-unc-119(+)] II; jfSi26[Psun-1::gfp cb-unc-119(+)] ced-3(n717) IV; sun-1(ok1282) V	This paper	UV147
C. elegans: [gfp::lmn-1^{S8A}]; [sun-1^{S10A}::gfp] ced-3(n717): lmn-1(tm1502) I; jfSi89[Plmn-1S(21,22,24,32,397,398, 403,405)A::GFPcb-unc-119(+)] II; jfSi49[Psun-1::gfp S(8, 12, 16, 24, 36, 43, 58, 62, 69, 78)A cb-unc-119(+)] ced-3(n717) IV; sun-1 (ok1282) V	This paper	UV148
C. elegans: [sun-1^{S10A}::gfp] ced-3(n717): jfSi49[Psun-1:: gfp S(8, 12, 16, 24, 36, 43, 58, 62, 69, 78)A cb-unc- 119(+)] ced-3(n717) IV; sun-1 (ok1282) V	This paper	UV146
Oligonucleotides		
For primers see Table S3	N/A	N/A
Recombinant DNA		
Destination vector pDESTttTi5605[R4-R3] for MOS insertion on Chromosome II (was a gift from Erik Jorgensen)	(Frøkjaer-Jensen et al., 2008)	pCFJ150<br>Addgene plasmid # 19329
Destination vector pCFJ178 - cxTi10882_MCS for cxTi10882 targetting region for MOS insertion on Chromosome IV (was a gift from Erik Jorgensen)	(Frøkjaer-Jensen et al., 2008)	pCFJ178<br>Addgene plasmid # 19331
Co-injection marker Pmyo-2::mCherry::unc-54utr (was a gift from Erik Jorgensen)	(Frøkjaer-Jensen et al., 2008)	pCFJ90<br>Addgene plasmid # 19327
Co-injection marker pGH8 - pRAB-3::mCherry:: unc-54utr (was a gift from Erik Jorgensen)	(Frøkjaer-Jensen et al., 2008)	pGH8<br>Addgene plasmid # 19359
MOS transposase Pglh-2::MosTase::glh-2utr (was a gift from Erik Jorgensen)	Frøkjaer-Jensen et al., 2008)	pJL43.1<br>Addgene plasmid # 19332
Entry clone for Gateway pDONR^{TM}221 (lmn-1 CDS was cloned in this vector- also versions with point mutations)	Multistite Gateway ® Kit, Thermo Fischer Scientific	Cat# 12537-023
pDONR^{TM} P4-P1R (the lmn-1 5' UTR with and without GFP was cloned in this vector)	Multistite Gateway ® Kit, Thermo Fischer Scientific	Cat# 12537-023
pDONRTM P2r-P3 (the lmn-1 3'UTR was cloned in this vector)	Multistite Gateway ® Kit, Thermo Fischer Scientific	Cat# 12537-023
Minigene from IDT for generating the lmn-18A containing the whole CDS of lmn-1(S21,22,24,32,397,398,403,405A)	This paper	N/A
Plmn-1_gfp::lmn-1_lmn-1 3'UTR in pCFJ150	This paper	N/A
Plmn-1_gfp::lmn-1(S8A)_lmn-1 3'UTR in pCFJ150	This paper	N/A
Plmn-1_gfp::lmn-1(S32,403A)_lmn-1 3'UTR in pCFJ150	This paper	N/A
Plmn-1_lmn-1(S32,403,470)_lmn-1 3'UTR in pCFJ150	This paper	N/A

(Continued on next page)

Continued		
REAGENT or RESOURCE	SOURCE	IDENTIFIER
Plmn-1_lmn-1_lmn-1 3'UTR in pCFJ150	This paper	N/A
Minigene from IDT for generating the SUN-1(10A) containing part of the CDS of SUN-1(8,12,16,24, 36,43,58,62,69,78A)	This paper	N/A
Psun-1::sun-1::eGFP::sun-1 3'UTR	Penkner et al., 2009	N/A
Peft-3::cas9-SV40_NLS::tbb-2 3'UTR was a gift from John Calarco	(Friedland et al., 2013)	Addgene plasmid # 46168
pRB1017 was a gift from Andrew Fire	(Arribere et al., 2014)	Addgene plasmid # 59936
Software and Algorithms		
ImageJ version 2.0.0<br>Plugins used: StackReg; Manual Tracking	NIH	https://imagej.nih.gov/ij/
SoftWorx Suite	Applied Precision	N/A
GraphPad Prism 6	GraphPad	N/A
Adobe Photoshop CC2015	Adobe	N/A
Adobe Illustrator CS6	Adobe	N/A
MaxQuant Software 1.5.6.0	MaxQuant	www.coxdocs.org
GPS SUMO SP 2.0	The CUCKOO Workgroup	http://sumosp.biocuckoo.org/online.php

CONTACT FOR REAGENT AND RESOURCE SHARING

Further information and requests for resources and reagents should be directed to and will be fulfilled by the Lead Contact, Verena Jantsch (verena.jantsch@univie.ac.at).

EXPERIMENTAL MODEL AND SUBJECT DETAILS

Worms were maintained at 20°C using standard techniques and media (Brenner, 1974), by growing them on Nematode Growth Medium agar plates seeded with *Eschericha coli* OP50. For all experiments, unless stated otherwise, young hermaphrodite adult worms were used. For this hermaphrodite L4 staged worms were pre-selected and kept at 20°C for 16-24 hours before conducting experiments.

gfp::*lmn-1* transgenic strains were generated using the MosSCI system (Frøkjaer-Jensen et al., 2008), a method to insert a single copy of a transgene into a defined chromosomal site.

Transgenes were inserted into chromosome II using the pCFJ150 plasmid as destination vector. In all cases, the N-terminus of LMN-1 was tagged and all strains were crossed into the deletion background after several out-crosses. The following regions were cloned using the Gateway® Multisite cloning system (Thermo Fischer) in the following order: 3924 bp of the endogenous 5' UTR of *lmn-1*; eGFP sequence; and the *lmn-1* genomic sequence including 1,321 bp of the endogenous 3′UTR.

To insert the GFP tag into the endogenous locus of *lmn-1*, we followed the method of Arribere et al. (Arribere et al., 2014) using the Cas9 plasmid from Friedland et al. (Friedland et al., 2013) and primers and sgRNAs specified in the Table S3.

METHOD DETAILS

Detergent Extraction Assay

Worms were dissected on a coverslip in M9 buffer containing 1 mM tetramisole, transferred to a 200 μl microcentrifuge tube and incubated in PBS containing 0.5% Triton X-100 for 5 min. All subsequent incubations were performed in the 200 μl tube. Triton was aspirated and dissected worms were fixed for 10 min in 2% formaldehyde in PBS containing 0.05% Tween-20 (PBS-T). Specimens were then washed three times in PBS-T (for 5 min). Control worms were subjected to the same procedure but omitting the Triton X-100 treatment. Worm strains expressing GFP::LMN-1 were immediately mounted in DAPI (4',6-diamidino-2-phenylindole)/Vectashield and images were acquired within 24 h to quantify endogenous GFP::LMN-1 intensity.

Worm strains not expressing GFP::LMN-1 were dissected and treated as described above. After the PBS-T washes, gonads were incubated for 30 min in blocking buffer (0.5% BSA in PBS-T) at room temperature, and then at 4°C overnight in primary antibody diluted in blocking buffer. Specimens were washed three times for 5 min in PBS-T before incubation for 2 h with the respective secondary antibodies at room temperature. After washing in PBS-T, specimens were mounted in DAPI/Vectashield and images were acquired within 24 h. After formaldehyde fixation, control worms not treated with 0.5% Triton were incubated for 10 min in 0.2% Triton in PBS-T to allow antibody penetration.

Immunofluorescence Analysis

Immunofluorescence was performed as previously described (Martinez-Perez and Villeneuve, 2005). Briefly, L4 hermaphrodites were incubated at 20°C for 24 h. Gonads were then dissected from young adults into 1× PBS, fixed in 1% formaldehyde for 5 min at room temperature and frozen in liquid nitrogen. After post-fixation in ice-cold methanol, non-specific binding sites were blocked by incubation in PBS containing 3% BSA for at least 20 min. Antibodies were diluted in blocking buffer and incubated overnight at 4°C (for primary antibodies) or 2 h at room temperature (for secondary antibodies).

Chromosome Spreads

Chromosome spreads were prepared as previously described (Pattabiraman et al., 2017) and analyzed using the LSM 710 Airyscan module. Briefly, gonads of young hermaphrodites were dissected in dissection buffer (0.1% Tween-20 in 80% DMEM) on a coverslip and 50 μl of spreading solution was added (32 μl of fixative (4% formaldehyde, 3.2% sucrose in water), 16 μl 1% lipsol in water, 2 μl 1% sarcosyl in water). The coverslips were dried for 2 hours at 37°C and washed in methanol at -20°C. Consecutively, coverslips were rehydrated by washing 3 times for 5 minutes each in PBS-T and unspecific binding sites blocked by incubating for 20 minutes in 1% BSA in PBS-T. Antibodies were diluted in blocking buffer and incubated overnight at 4°C (for primary antibodies) or 2 h at room temperature (for secondary antibodies).

FISH Analysis

The FISH protocol was based on a published protocol (Silva et al., 2014). Dissected gonads were fixed in 4% paraformaldehyde in egg buffer for 2 min at room temperature and then stored in methanol at −20°C. Slides were then incubated in methanol at room temperature for 20 min, followed by 1 min washes in 50% methanol and 1× SCCT and dehydration by sequential immersion in 70%, 90% and 100% ethanol (3 min each). Hybridization mixture containing 10.5 μl FISH buffer (1 ml 20× SCCT, 5 ml formamide, 1 g dextran sulphate, 4 ml H_2O) and 2.5 μl labeled probe was added to air-dried slides. The FISH probe for the 5S rDNA locus (chromosome V) was made by labeling 1 μg DNA with the DIG (Digoxigenin)-nick translation kit (Roche). After addition of EDTA, the probe was incubated at 65°C for 10 min. Oligos used for FISH probe amplification are specified in Table S3. Slides were incubated at 37°C overnight in a humidified chamber and then washed twice (20 min) at 37°C in 50% formamide, 2X SCCT and 1X 10% Tween. After three washes in 2X SCCT at room temperature, samples were blocked for 1 h in 2X SCCT containing 1X BSA (w/v). Slides were then incubated in secondary anti-biotin antibody diluted in 2X SCCT (1:500) for 2 h at room temperature, followed by three washes in 2X SCCT, and then stained with 1 ng/ml DAPI and mounted in Vectashield.

SYTO-12 Staining

Young adults (24 h post-L4 stage) were soaked in 33 μM SYTO-12 in PBS for 2–3 h at 20°C in the dark, transferred to unseeded NGM (nematode growth medium) plates for 30–60 min and then mounted. SYTO-12 positive cells were scored within the germline using an epifluorescence microscope equipped with a 40× or 63× oil immersion objective lens.

Image Acquisition

Images were acquired using either a DeltaVision system equipped with 60×/1.42 and 100×/1.40 oil immersion objective lenses and a complementary softWORx software package or a Zeiss LSM 710 system equipped with an Airyscan detection module and a 100×/1.46 oil immersion objective lens. Unless otherwise stated, images are maximum projections of entire nuclei. Images acquired with the DeltaVision where deconvolved using the softWORx deconvolution algorithm. Maximum intensity projections of deconvolved images were generated using Fiji/ImageJ after background subtraction using a rolling ball radius of 50 pixel. Where specified, images of gonads consist of multiple stitched images. This is necessary due to the size limitation of the field of view at high magnifications. Stitching of images to build up entire gonads was performed manually in Adobe Photoshop. Levels of stitched images were adjusted to each other in Adobe Photoshop to correct for auto-adjustment settings of the microscope.

Live Imaging of Worms for Monitoring of Chromosome Movement

For analyzing the movement of SUN-1 and HIM-8 aggregates, worms were treated as previously described (Baudrimont et al., 2010). Adult hermaphrodites were pre-selected at the L4 stage 14–16 h before filming and mounted on 2% agarose pads in M9 containing 1 mM tetramisole; coverslips were sealed with melted petroleum jelly. Images were acquired as 0.8 μm thick optical sections every 5 s for 5 min. Data was analyzed using ImageJ (NIH) with StackReg and Manual Tracking plugins.

Live Imaging of Worms for Monitoring of the First Embryonic Division

For live cell imaging of mitotic nuclear envelope breakdown, embryos were filmed without compression (Monen et al., 2005) on a Yokogawa CSU X1-A1 spinning disk confocal mounted on a Zeiss Axio Observer Z1 inverted microscope equipped with a 63x 1.25NA Plan-Neofluar lens, 100mW 488nm and 561nm solid-state lasers, and Evolve EM512 back-illuminated EM-CCD camera and controlled by VisiView software (Visitron Systems). 8x1 μm GFP/mCherry z-series as well as single plane DIC images were acquired every 30s from meiosis II until the end of mitosis, using low laser illumination to minimize photobleaching/photodamage. Images presented are maximum intensity projections of most of the entire nuclear/spindle volume prepared in Image J. Nuclear envelope breakdown was defined as the time point at which free nuclear histone::mCherry signal had fully equilibrated with the cytoplasm.

FRAP Analysis of the Nuclear Lamina in Living Worms

For FRAP analysis, worms were selected and mounted as described above for the live imaging. For FRAP analysis the above specified spinning disk confocal microscope was used. The GFP::LMN-1 signal was measured on single planes at each time point using Metamorph software. For details on the signal quantification please see the "Quantification and Statistical Analysis" section.

Electron Microscopy

High-Pressure Freezing

Young adult worms were transferred to a drop of 10% BSA in M9 buffer. Gonads were extracted with a syringe and pipetted into the 100 μm recess of a 3 mm freezing platelet; excess liquid was carefully removed with filter paper. The flat side of a second platelet was placed on top as a lid. Samples were cryo-immobilized using an EM HPM100 high-pressure freezing machine (Leica Microsystems) and stored in liquid nitrogen until freeze substitution.

Freeze Substitution and Imaging

Samples were processed by freeze substitution as previously described (Stigloher et al., 2011), except that Epon was used for embedding and polymerization was done at 60°C for 72 h. After embedding, ultrathin sections were cut and transferred to copper grids. The sections were contrasted in 2% uranyl acetate in H_2O for 15 minutes at room temperature, followed by incubation in lead citrate for 10 minutes. Grids were washed before image acquisition with a JEOL JEM-2100 operated at 200kV. Also see Table S2 for criteria for the choice of nuclei analyzed.

Proteomic Analysis

Sample Processing

Worms were grown and nuclei were isolated as previously described (Silva et al., 2014). Frozen nuclear pellets were solubilized in 250 μl 8 M urea in 20 mM HEPES (pH 8.0) containing phosphatase inhibitors (100 mM sodium orthovanadate, 500 mM sodium fluoride, 1 M glycerol phosphate, 250 mM disodium pyrophosphate). Samples containing 300 μg protein were reduced in 10 mM dithiothreitol and alkylated in 50 mM iodoacetamide. Samples were then diluted with 20 mM HEPES buffer (pH 8.0) to a final concentration of 2 M urea and trypsin (V5280, Promega) at a 1:50 ration to protein was added and incubated overnight at 37°C. Samples were then acidified by adding trifluoroacetic acid (TFA) to a final concentration of 1%. For total protein analysis, a 50 μg protein sample was desalted using C18 spin tips (TT2C18.96, Glygen) and peptides were eluted in 60% acetonitrile containing 0.1% formic acid and dried using a centrifugal vacuum drier. The remaining 200 μg sample was de-salted using solid phase extraction with 10 mg cartridges (Oasis HLB, Waters) according to the manufacturer's instructions.

Peptides were eluted with 1 M glycolic acid in 80% ACN and 5% TFA and phospho-enriched using a TiO_2-based method (Casado et al., 2014). Briefly, eluent volume was adjusted to 1 ml with 1 M glycolic acid solution and then incubated with 25 mg TiO_2 (50% slurry in 1% TFA) for 5 min at room temperature. After incubation for 5 min with mixing, the TiO_2/peptide mixture was packed into empty spin tips by centrifugation and the TiO_2 layer was sequentially washed with 1 M glycolic acid, 100 mM ammonium acetate in 25% ACN and 10% ACN. Phosphopeptides were eluted by four sequential additions of 50μl 5% NH_4OH, clarified by centrifugation at 18,000 g for 2 min and transferred to new tubes. Samples were snap frozen on dry ice and dried by vacuum centrifugation.

LC-MS/MS

Phospho-enriched samples and desalted and digested total protein samples were dissolved in 0.1% TFA by shaking (1200 rpm) for 30 min, sonication in an ultrasonic water bath for 10 min and centrifugation (14,000 rpm, 5°C) for 10 min. LC-MS/MS analysis was carried out in technical duplicates. Total protein (1 μg) was separated on an Ultimate 3,000 RSLCnano liquid chromatography system (Thermo Scientific) coupled to an Orbitrap Velos mass spectrometer (Thermo Scientific) via a Dionex nano-electrospray source. Phosphopeptide solutions were injected onto a trap column (Acclaim PepMap 100 C18; 100 μm × 2 cm) for desalting and concentration at 8 μl/min in 2% ACN and 0.1% TFA, and eluted onto an on-line analytical column (Acclaim PepMap RSLC C18, 75 μm × 50 cm) at a flow rate of 250 nl/min. Peptides were separated using a 120 min gradient (4–25% buffer B (80% acetonitrile, 0.1% formic acid) for 90 min, followed by 25–45% buffer B for 30 min, followed by column conditioning and equilibration). Eluted peptides were analyzed by mass spectrometry in positive polarity and data-dependent acquisition mode. The 10 most abundant ions were selected for fragmentation from an initial MS1 survey scan at a resolution of 30,000, followed by collision-induced dissociation. Automatic gain control targets for MS1 and MS2 scans were set to 1×10^6 and 3×10^4 for maximum injection times of 500 ms and 100 ms, respectively. A survey scan *m/z* range of 350–1500 was used, with multistage activation enabled, normalized collision energy set to 35%, charge state screening enabled with +1 charge states rejected, and a minimal fragmentation trigger signal threshold of 500 counts. For details on the raw data processing please see the "Quantification and Statistical Analysis" section.

QUANTIFICATION AND STATISTICAL ANALYSIS

Quantification of RAD-51 Foci

The gonad was divided into seven equal zones and the number of RAD-51 foci per nucleus was counted in each zone. Graphs show the percentage of nuclei corresponding to each of the following data groups: 0 foci, 1 focus, 2–3 foci, 4–6 foci, 7–12 foci, >12 foci and stretches (defined as a continuous signal unresolvable as single foci).

Quantification of X-Chromosome Pairing

The gonad was divided into seven equal zones and the number of nuclei containing one or two HIM-8 foci was counted in each zone. Graphs show the percentage of nuclei with paired HIM-8 signal in each zone.

Quantification of SC Assembly

The gonad was divided into 10 equal zones and the number of nuclei with full colocalization of HTP-3 (chromosome axes) with SYP-1 (SC central element) in each zone was recorded. Graphs show the percentage of nuclei with complete synapsis in each zone.

Quantification of Fluorescence Signal Intensities

Fluorescence intensity was measured on undeconvolved single-plane images in Fiji/ImageJ.

Single confocal images were used to obtain ratios for lamin intensity at the nuclear rim and nuclear interior. Lamin intensity was measured in circular regions of interest covering either the entire nuclear lamina or only the nuclear interior after normalizing intensity values to overall nuclear fluorescence and subtracting background fluorescence. Image processing was done in Fiji/ImageJ and Adobe Photoshop.

Quantification of FRAP Signal Intensities

Two concentric regions were drawn around the nuclear lamina, a smaller one encompassing the lamina at the bleached region, and a larger one including the lamina plus its surrounding area. The integrated GFP intensity was then calculated by subtracting the mean fluorescence intensity in the area between the two regions of interest (mean background) from the mean intensity in the smaller region and multiplying by the area of the smaller region. Measurements done after photobleaching were normalized to the mean intensity of measurements made up to 120s before photobleaching. Data points in the graphs are the mean of the normalized GFP intensity measurements collected during the 200s interval centered on that point; except for time points -10s and 0s where the means are calculated within 20s interval. Error bars indicate the 90% confidence interval for the mean.

Raw Data Processing of Proteomic MS Data

Data was processed using the MaxQuant software platform (v1.5.6.0), with UniProt *C. elegans* database (version 20170418, number of entries: 27,510) searching by the built-in Andromeda search engine. A reverse decoy database approach was used at a false discovery rate of 1% for peptide spectrum matches. Search parameters were: maximum missed cleavages set to 2; fixed modification of cysteine carbamidomethylation and variable modifications of methionine oxidation; protein N-terminal acetylation; and serine, threonine and tyrosine phosphorylation. Label-free quantification was enabled with a minimum ratio count of 2. The 'match between runs' function used match and alignment time limits of 1 and 20 min, respectively.

For total protein samples, search parameters included variable modifications: methionine oxidation, protein N-terminal acetylation, asparagine deamidation and N-terminal glutamine cyclization to pyroglutamate.

Statistical Analysis

Statistical analyses were performed in GraphPad Prism6. Datasets were tested for normal distribution; depending on outcome, populations were tested for significant differences using the two-tailed Student's *t*-test, Chi Square test or Mann–Whitney *U*-test, as appropriate for each dataset. Generally, *p-values* less than or equal to 0.05 were considered 'significant' (*), less than 0.01 'very significant' (**) and less than 0.001 'highly significant' (***). Statistical details can be found within the respective figure legends for datasets represented in figures. For datasets discussed only within the text, statistical details are described within the results.

To calculate the speed of SUN-1 aggregate movement, outliers were identified using the ROUT method in GraphPad Prism6 and the desired maximum false rate was set to 1%.

Developmental Cell, Volume *45*

Supplemental Information

Transient and Partial Nuclear Lamina Disruption Promotes Chromosome Movement in Early Meiotic Prophase

Jana Link, Dimitra Paouneskou, Maria Velkova, Anahita Daryabeigi, Triin Laos, Sara Labella, Consuelo Barroso, Sarai Pacheco Piñol, Alex Montoya, Holger Kramer, Alexander Woglar, Antoine Baudrimont, Sebastian Mathias Markert, Christian Stigloher, Enrique Martinez-Perez, Alexander Dammermann, Manfred Alsheimer, Monique Zetka, and Verena Jantsch

Supplemental Information

Figure S1

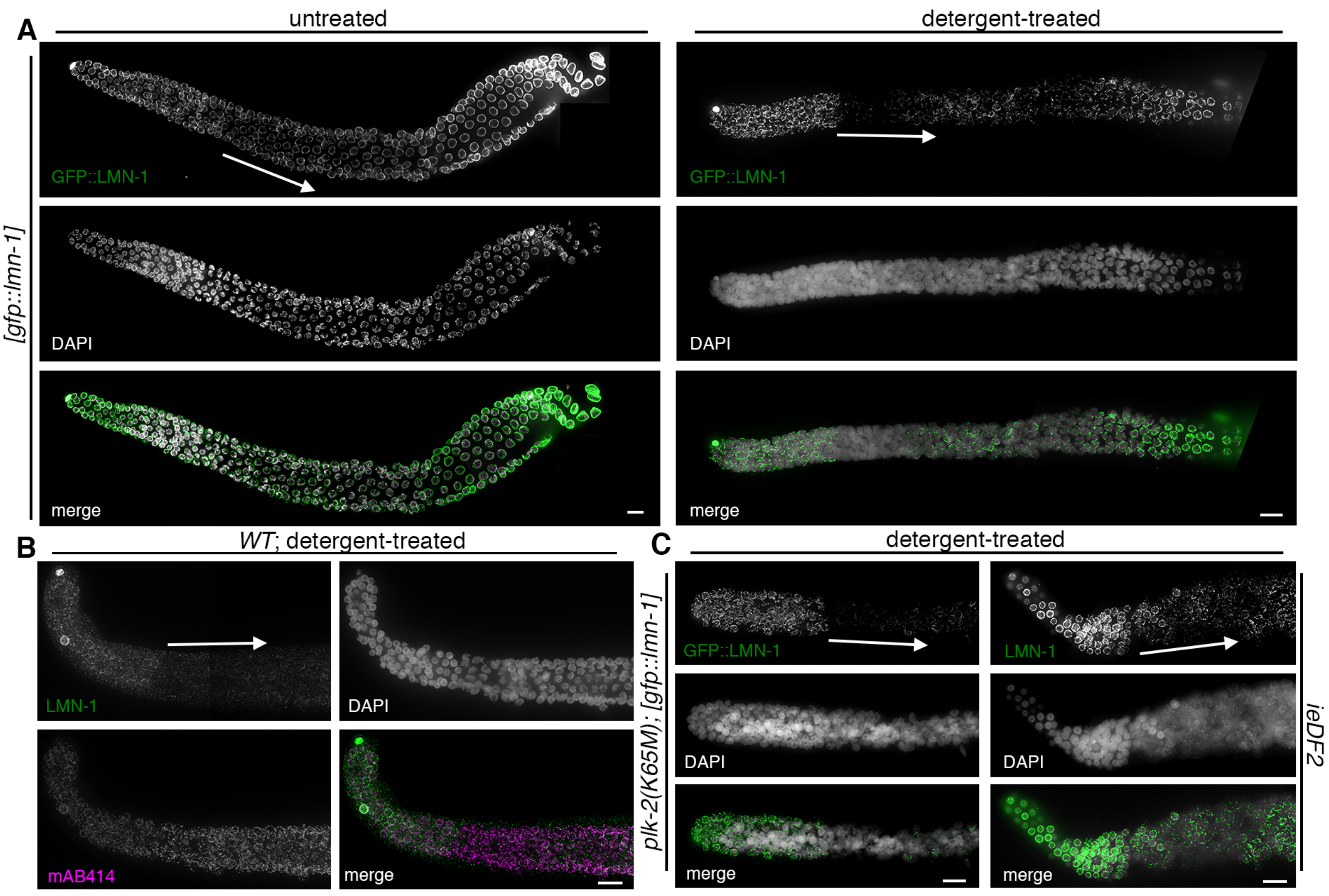

Figure S2

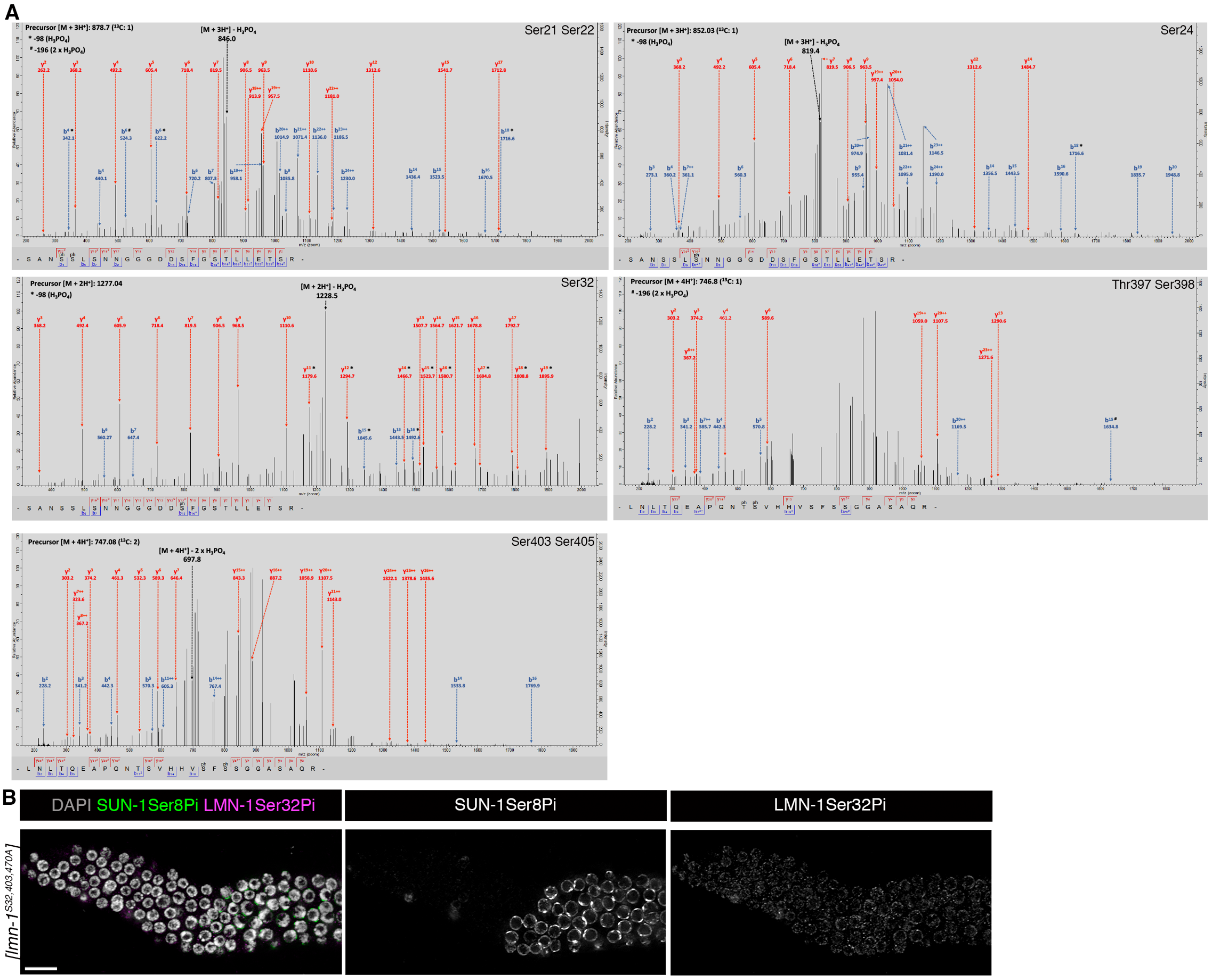
A
Ser21 Ser22
Ser24
Ser32
Thr397 Ser398
Ser403 Ser405
B
DAPI SUN-1Ser8Pi LMN-1Ser32Pi
SUN-1Ser8Pi
LMN-1Ser32Pi
[lmn-1S32,403,470A]

Figure S3

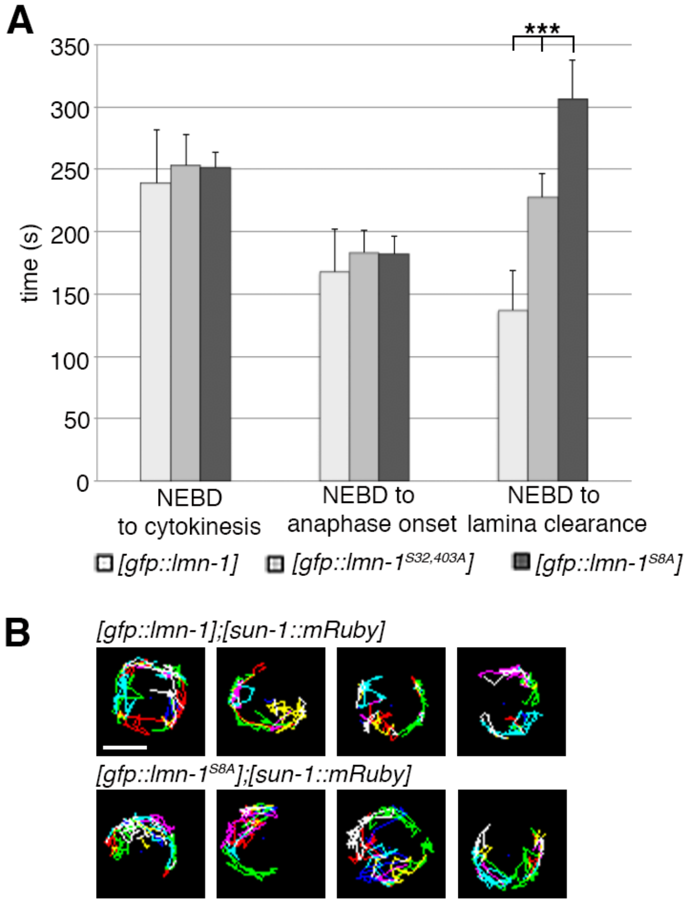
A
350
300
250
200
150
100
50
0
time (s)
***
NEBD
to cytokinesis
NEBD to
anaphase onset
NEBD to
lamina clearance
[gfp::lmn-1]
[gfp::lmn-1S32,403A]
[gfp::lmn-1S8A]
B
[gfp::lmn-1];[sun-1::mRuby]
[gfp::lmn-1S8A];[sun-1::mRuby]

Figure S4

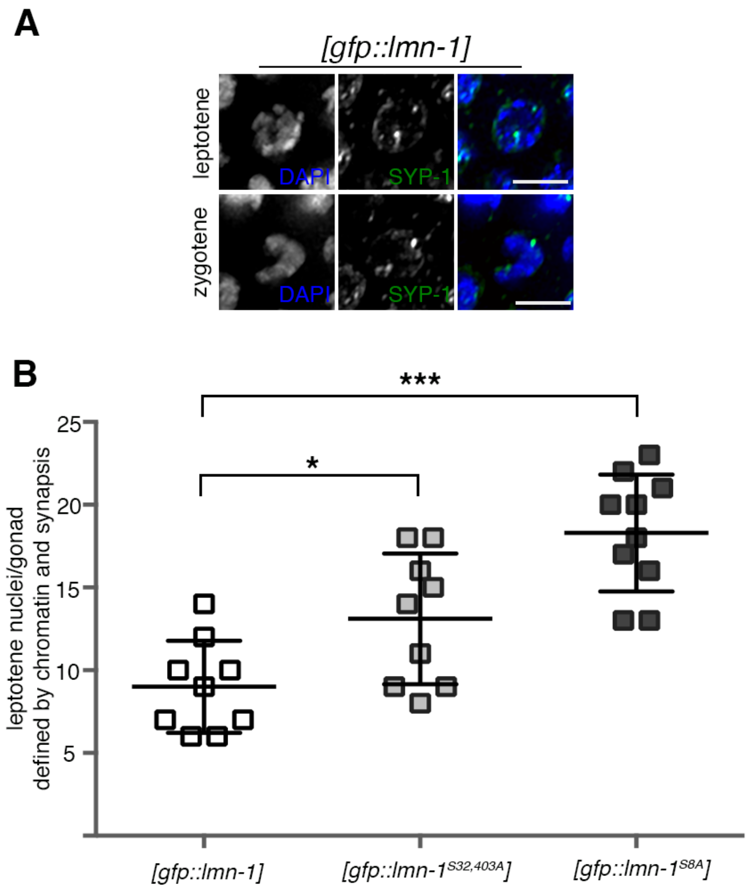
A
[gfp::lmn-1]
leptotene
zygotene
DAPI
SYP-1
DAPI
SYP-1
B
***
*
leptotene nuclei/gonad defined by chromatin and synapsis
25
20
15
10
5
[gfp::lmn-1]
[gfp::lmn-1S32,403A]
[gfp::lmn-1S8A]

Figure S5

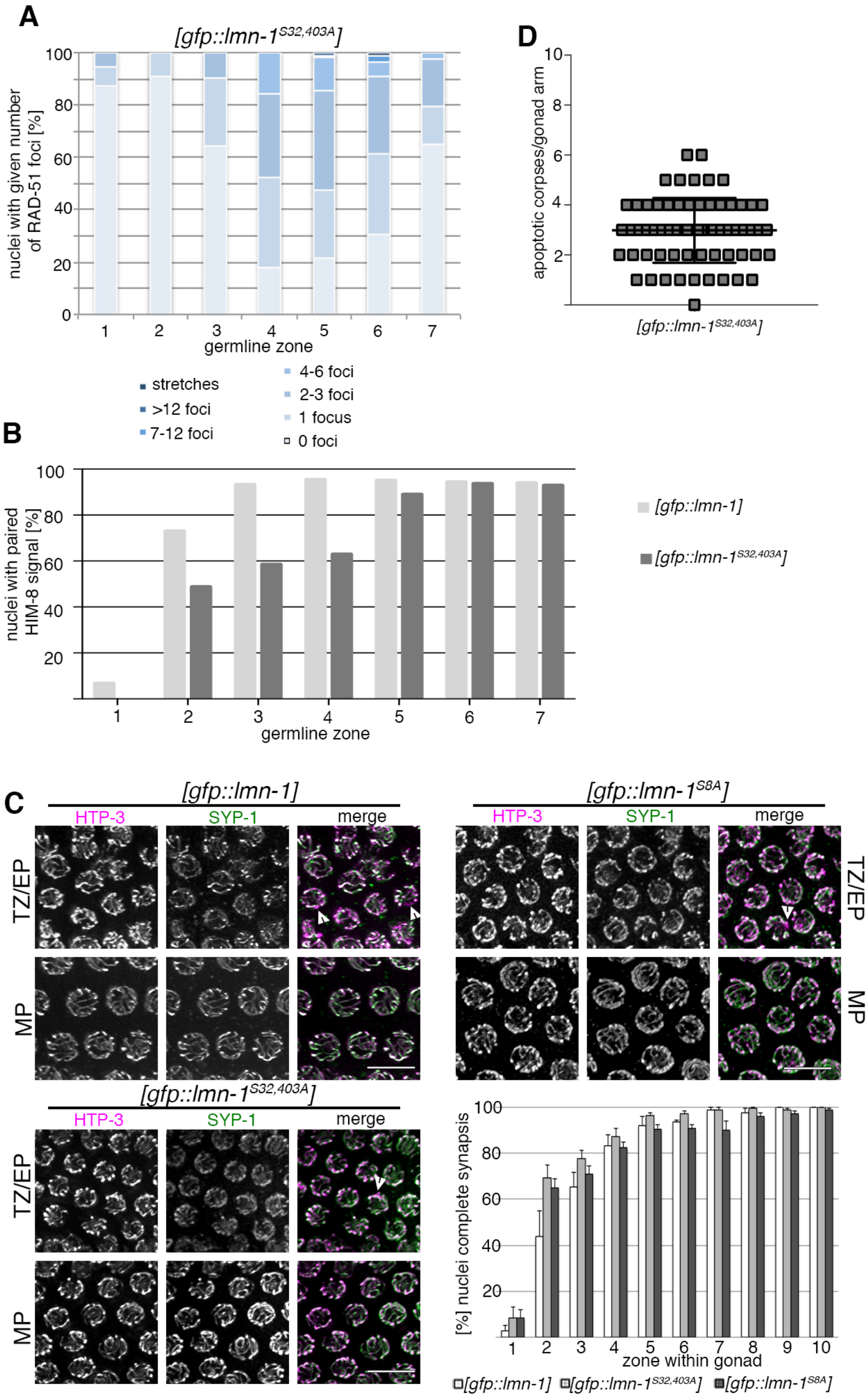
A
[gfp::lmn-1S32,403A]
nuclei with given number of RAD-51 foci [%]
100
80
60
40
20
0
1 2 3 4 5 6 7
germline zone
stretches
>12 foci
7-12 foci
4-6 foci
2-3 foci
1 focus
0 foci
D
apoptotic corpses/gonad arm
10
8
6
4
2
[gfp::lmn-1S32,403A]
B
nuclei with paired HIM-8 signal [%]
100
80
60
40
20
1 2 3 4 5 6 7
germline zone
[gfp::lmn-1]
[gfp::lmn-1S32,403A]
C
[gfp::lmn-1]
HTP-3
SYP-1
merge
TZ/EP
MP
[gfp::lmn-1S8A]
HTP-3
SYP-1
merge
TZ/EP
MP
[gfp::lmn-1S32,403A]
HTP-3
SYP-1
merge
TZ/EP
MP
[%] nuclei complete synapsis
100
80
60
40
20
1 2 3 4 5 6 7 8 9 10
zone within gonad
[gfp::lmn-1]
[gfp::lmn-1S32,403A]
[gfp::lmn-1S8A]

Figure S6

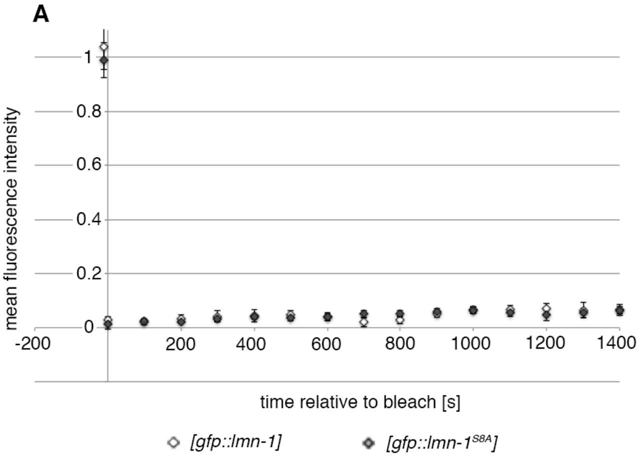
A
mean fluorescence intensity
1
0.8
0.6
0.4
0.2
0
-200
200
400
600
800
1000
1200
1400
time relative to bleach [s]
[gfp::lmn-1]
[gfp::lmn-1^S8A]

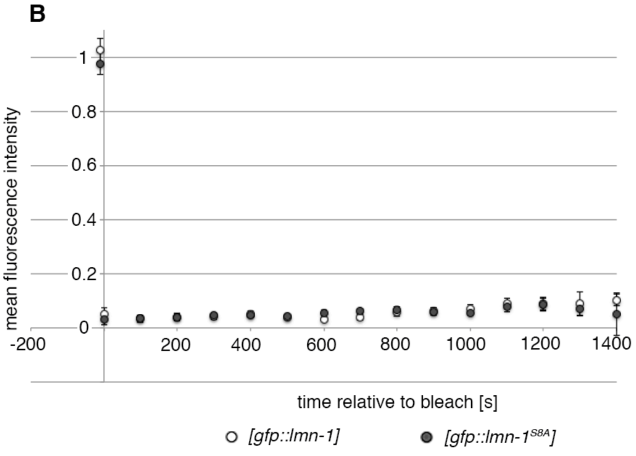
B
mean fluorescence intensity
1
0.8
0.6
0.4
0.2
0
-200
200
400
600
800
1000
1200
1400
time relative to bleach [s]
[gfp::lmn-1]
[gfp::lmn-1^S8A]

Figure S1

Decreased detergent resistance is specific to the lamina of early meiotic nuclei and is less prominent in *ieDF2* mutants. Related to Figure 1. (A) GFP::LMN-1 localization in untreated and detergent-treated wild-type gonads. The LMN-1 localization pattern in detergent-treated gonads is highly reproducible, reflecting changing detergent sensitivity as cells progress through the different prophase I stages. (B) Nuclear pore (mAB414) staining and LMN-1 localization pattern in detergent-treated wild-type gonads. (C) GFP::LMN-1 localization and LMN-1 immunofluorescence in detergent-treated *plk-2(K65M)* and *ieDF2* gonads. Arrows indicate the meiotic entry/transition zone; scale bars, 10 µm. Gonads shown in (A-C) consist of multiple maximum projection images stitched together to show larger sections of the gonads.

Figure S2

Identification of meiotic LMN-1 phosphorylation sites. Related to Figure 2. (A) Selected MS/MS fragmentation spectra showing phosphorylation sites on phosphopeptides corresponding to LMN-1 (Q21443) residues 18–42 (SANSSLSNNGGGDDSFGSTLLETSR) and 387–413 (LNLTQEAPQNTSVHHVSFSSGGASAQR). Annotated peaks correspond to ion species with a mass difference of −98 (*) or −196 (#), resulting from the loss of one or two phosphate moieties, respectively. Note that in the spectra showing Thr397 and Ser398 phosphorylation, the mass of ions Y19, Y20 and Y23 correspond to intact doubly phosphorylated fragments. (B) The LMN-1Ser32pi antibody specifically recognizes phospho-Ser32 LMN-1. LMN-1Ser32pi and SUN-1Ser8pi staining in transition zone of a mutant expressing Ser32A LMN-1. Scale bar, 10 µm. Gonads shown in (B) consist of multiple maximum projection images stitched together to show larger sections of the gonads.

Figure S3

Mutants expressing non-phosphorylatable LMN-1 have delayed lamina clearance after mitotic nuclear envelope breakdown (NEBD). Related to Figure 3. (A) Kinetics of the first mitotic embryonic division, showing the average periods between selected

hallmark mitotic events in the wild type and in two mutants expressing non-phosphorylatable LMN-1. *[gfp::lmn-1]*, *n* = 8 embryos; *[gfp::lmn-1^{S32,403A}]*, *n* = 6 embryos; *[gfp::lmn-1^{S8A}]*, *n* = 6 embryos. For timing from NEBD to lamina clearance: *P* = 9.04×10^{-5} (***) for *[gfp::lmn-1]* vs *[gfp::lmn-1^{S32,403A}]*, 8.05×10^{-7} (***) for *[gfp::lmn-1]* vs *[gfp::lmn-1^{S8A}]* and 6.77×10^{-4} (***) for *[gfp::lmn-1^{S32,403A}]* vs *[gfp::lmn-1^{S8A}]*. *P* > 0.05 (ns) not indicated. *P* values were calculated using the two-tailed Student's *t*-test. Error Bars represent SD. (B) Additional displacement tracks of wild type and non-phosphorylatable *lmn-1* mutants. Despite the significantly reduced velocity of SUN-1::mRuby aggregates, they show comparable exploration of the nuclear surface. Scale bar, 5 µm.

Figure S4

SYP-1 immunofluorescence (synapsis marker) shows that leptotene nuclei accumulate in the gonads of mutants expressing non-phosphorylatable LMN-1. Related to Figure 4. (A) Leptotene nuclei are characterized by spherical, non-polarized chromatin with aggregates or small stretches of SYP-1. Zygotene nuclei also show aggregates or stretches of SYP-1 but are distinguished by their non-spherical, polarized chromatin. Scale bar, 5 µm. (B) Quantification of leptotene nuclei in *[gfp::lmn-1]*, *[gfp::lmn-1^{S32,403A}]* and *[gfp::lmn-1^{S8A}]* gonads. *[gfp::lmn-1]*, *n* = 9 gonads; *[gfp::lmn-1^{S32,403A}]*, *n* = 9 gonads; *[gfp::lmn-1^{S8A}]*, *n* = 10 gonads. *P* = 0.021 (*), *P* < 0.0001 (***). *P* values were calculated using the two-tailed Student's *t*-test. Scatter plots indicate mean and SD.

Figure S5

Meiotic chromosome axis formation and synapsis are normal in all mutants expressing non-phosphorylatable LMN-1; for other meiotic markers, *[gfp::lmn-1^{S32,403A}]* has an intermediate phenotype between *[gfp::lmn-1]* and *[gfp::lmn-1^{S8A}]*. Related to Figure 5. (A) Kinetics of RAD-51 localization in the *[gfp::lmn-1^{S32,403A}]* germline (addition to Fig. 5B). *[gfp::lmn-1^{S32,403A}]*, *n* = 3 gonads. Wild-type values are identical to those in Fig. 5B. (B) Kinetics of X-chromosome pairing in the *[gfp::lmn-1^{S32,403A}]* germline (addition to Fig. 5C). *[gfp::lmn-1^{S32,403A}]*, *n* = 5 gonads. Wild-type

values are identical to those in Fig. 5C. *P* values were calculated using the Chi square test and equal to 0.0028 (**), <0.0001(****), <0.0001 (****), <0.0001 (****), 0.1564 (ns), 0.9116 (ns), 0.5281 (ns) for each germline zone, respectively. (C) Kinetics of meiotic synapsis formation. HTP-3 immunofluorescence marks both unsynapsed and synapsed chromosome axes; SYP-1 localizes only to synapsed chromosomes. Bottom right panel, proportion of nuclei in each zone showing complete SYP-1/HTP-3 colocalization (indicating completed synapsis). TZ/EP, transition zone/early pachytene cells; MP, mid-pachytene cells; arrowheads indicate regions with incomplete synapsis. *[gfp::lmn-1]*, *n* = 4 gonads; *[gfp::lmn-1^{S32,403A}]*, *n* = 7 gonads; *[gfp::lmn-1^{S8A}]*, *n* = 7 gonads. Error bars indicate SEM. Scale bars, 5µm. (D) Quantification of SYTO-12 positive corpses per gonad arm in the *[gfp::lmn-1^{S32,403A}]* mutant (addition to Fig. 5E). *[gfp::lmn-1^{S32,403A}]*, *n* = 67 gonads; *P* > 0.05 (ns) compared with *[gfp::lmn-1]* worms. *P* values were calculated using the Mann–Whitney *U*-test. Scatter plots indicate mean and SD.

Figure S6

FRAP analysis of LMN-1 in nuclei in the mitotic and the meiotic zones show no significant recovery. Related to Figure 3. (A) FRAP analysis of nuclei in the mitotic zone in *[gfp::lmn-1]* and *[gfp:lmn-1^{S8A}]* worms. The normalized mean intensity of GFP::LMN-1 is shown. Error bars indicate 90% confidence intervals. *[gfp:lmn-1], n* = 3 nuclei; *[gfp::lmn-1^{S8A}]*, *n* = 5 nuclei. (B) FRAP analysis of meiotic nuclei in transition zone in *[gfp::lmn-1]* and *[gfp:lmn-1^{S8A}]* worms. The normalized mean intensity of GFP::LMN-1 is shown. Error bars indicate 90% confidence intervals. *[gfp:lmn-1], n* = 3 nuclei; *[gfp::lmn-1^{S8A}]*, *n* = 3 nuclei.

Table S1: Viability and brood size of strains used in this study. Related to experimental models in STAR Methods.

Strain	% Viability +/- sd	Brood size +/- sd	n
lmn-1(tm1502);[gfp::lmn-1]	99.4% +/- 0.8	223.6 +/- 30.9	18
lmn-1(tm1502); [gfp::lmn-1^{S32,403A}]	99.2% +/- 0.7	230.1 +/- 39.5	18
lmn-1(tm1502); [gfp::lmn-1^{S8A}]	99.3% +/- 0.6	221.5 +/- 43.2	18
lmn-1(tm1502); [gfp::lmn-1]; ced-3(n717)	95.4% +/- 6.5	161.1 +/- 38.6	14
lmn-1(tm1502); [gfp::lmn-1^{S32,403A}]; ced-3(n717)	89.7% +/- 21.2	127.7 +/- 41.7	14
lmn-1(tm1502); [gfp::lmn-1^{S8A}]; ced-3(n717)	74.3% +/- 21.4	134.3 +/- 56.3	14
[sun-1^{S10A}::gfp]; sun-1(ok1282)[1]	55.29% +/- 13.3	135.15 +/- 29.6	20
lmn-1(tm1502);[gfp::lmn-1]; [sun-1::gfp]; sun-1(ok1282)	99.1% +/- 1.7	227.4 +/- 31.2	20
lmn-1(tm1502);[gfp::lmn-1]; [sun-1^{S10A}::gfp]; sun-1(ok1282)	57.6% +/- 18.4	158.2 +/- 45.8	20
lmn-1(tm1502); [gfp::lmn-1^{S8A}]; [sun-1^{S10A}::gfp]; sun-1(ok1282)	50.69% +/- 23.5	134.8 +/- 42.4	17
[sun-1^{S10A}::gfp] ced-3(n717); sun-1(ok1282)	46.9% +/- 13.8	128.2 +/- 47.2	13
lmn-1(tm1502);[gfp::lmn-1]; [sun-1::gfp] ced-3(n717); sun-1(ok1282)	95.5% +/- 0.9	156.0 +/- 48.5	7

[1] This is a single copy integration line on chromosome IV. We obtained the integration line with the same transgene on chromosome II with 88% viability. This difference is likely due to lower expression levels of the transgene when integrated on chromosome IV.

lmn-1(tm1502); [gfp::lmn-1^{S8A}]; [sun-1^{S10A}::gfp] ced-3(n717); sun-1(ok1282)	49.2 +/- 9.7	101.6 +/- 50.7	10
lmn-1(jf98[GFP::lmn-1])	68% +/- 15.97	182.5% +/- 63.68	10

Table S2: Parameters used for the EM analysis. Related to Figure 5.

cell stage	Nuclear diameter in EM [µm]	Cellular characteristics
mitotic	not analyzed	no polarised nucleolus
early prophase (transition zone)	2.5-3.5	polarised nucleolus<br>germline granules present
later prophase<br>(mid- late pachytene)	above 4.3	Frequent extended SCs<br>Ref. *

* Rog, O. et al; 2017 – mid/late prophase nuclei with intact, extended synapsis have an approx. diameter of 4.9 µm i.e. larger than what we analyzed

Table S3. Oligonucleotides used in this study. Related to STAR Methods.

REAGENT or RESOURCE	SOURCE	IDENTIFIER
Oligonucleotides		
Forward primer for cloning the lmn-1 5' UTR into the P4-P1R entry vector<br>5' GGGGACAACTTTGTATAGAAAAGTTGGGGAAAA ACTACCGCAAAAAACCA 3'	This paper	N/A
Forward primer for bridging the GFP with the 5' UTR<br>5' GCAGCGAGAAAAATGGTGAGCAAGGGCGAG 3'	This paper	N/A
Reverse primer for cloning the lmn-1 5'UTR merged with the GFP into the P4-P1R entry vector<br>5' GGGGACTGCTTTTTTGTACAAACTTGGCTTGTACAGCTCGTCGTCCATG 3'	This paper	N/A
Reverse primer for cloning the lmn-1 5' UTR into the P4-P1R entry vector<br>5' GGGGACTGCTTTTTTGTACAAACTTGG TTTTCTCGCTGCTTTCTGAAGAG	This paper	N/A
Forward primer for cloning lmn-1 CDS into the pDONR 221 vector<br>5' GGGGACAAGTTTGTACAAAAAAGCAGGCTCC ATGTCATCTCGTAAAGGTACTCGTA 3'	This paper	N/A
Reverse primer for cloning lmn-1 CDS into the pDONR 221 vector<br>5' GGGGACCACTTTGTACAAGAAAGCTGG GTCTTACATGATGGAACAACGATCGG 3'	This paper	N/A
Forward primer for cloning the lmn-1 3' UTR into the P2r-P3 entry vector<br>5' GGGGACAGCTTTCTTGTACAAAGTGGGG TTGAGAGTCGATATTACATCAATCC 3'	This paper	N/A
Reverse primer for cloning the lmn-1 3' UTR into the P2r-P3 entry vector<br>5' GGGGACAACTTTGTATAATAAAGTTG CAACTTGATGTCCTGCCGAG 3'	This paper	N/A
Forward primer for introducing the S32A point mutation<br>5' AGGAGGCGACGATGCTTTGT3'	This paper	N/A
Reverse primer for introducing the S32A point mutation<br>5' ACAAAGCATCGTCGCCTCCT 3'	This paper	N/A
Forward primer for introducing the S403A point mutation<br>5' TCATCACGTCGCTTTTTCATCC 3	This paper	N/A
Reverse primer for introducing the S403A point mutation<br>5' GGATGAAAAAGCGACGTGATGA 3'	This paper	N/A
Forward primer for introducing the S470A point mutation<br>5' AAGAAGAACAAGCTATCGGAGGAT 3'	This paper	N/A

Reverse primer for introducing the S470A point mutation<br>5’ ATCCTCCGATAGCTTGTTCTTCTT 3’	This paper	N/A
Forward primer for generating sgRNA1 for tagging lmn-1 with GFP<br>*5’* TCTTGGAGAGATGTAAGAGAAGAG *3’*	This paper	N/A
Reverse primer for generating sgRNA1 for tagging lmn-1 with GFP<br>5’ AAACCTCTTCTCTTACATCTCTCC 3’	This paper	N/A
Forward primer for generating sgRNA2 for tagging lmn-1 with GFP<br>5’ TCTTGTCTGAAGAGTTAATTATAT 3’	This paper	N/A
Reverse primer for generating sgRNA2 for tagging lmn-1 with GFP<br>5’ AAACATATAATTAACTCTTCAGAC 3’	This paper	N/A
Forward primer for generating sgRNA3 for tagging lmn-1 with GFP<br>5’ TCTTGGAAAAATGTCATCTCGTAA 3’	This paper	N/A
Reverse primer for generating sgRNA3 for tagging lmn-1 with GFP<br>5’ AAACTTACGAGATGACATTTTTCC 3’	This paper	N/A
Forward primer for generating the ds repair template for the GFP tagging of lmn-1<br>5’ CAAGAAATATCAAAATGTTGAGTCACGTTCGTGTGG CGCCTATGACGTTTTCTCTCTTCTCTTACATCTCTCTT TGTCTATTTCTGAAACTAAATTCAATATAATTAACTCTT CAGAAAGCAGCGAGAAAAATGGTGAGCAAGGGCGAG 3’	This paper	N/A
Reverse primer for generating the ds repair template for the GFP tagging of lmn-1<br>5’ GCGACGAATTCGCTGAGCGCTCTAGCGTAACAATACGAGAACT ACGAGTACCTTCACGAGGTGACATCTTGTACAGCTCGTCCATGC 3’	This paper	N/A
Forward primer for FISH probe amplification<br>5’ TACTTGGATCGGAGACGGCC 3’	(Silva et al., 2014)	N/A
Reverse primer for FISH probe amplification<br>5’ CTAACTGGACTCAACGTTGC 3’	(Silva et al., 2014)	N/A
